# Measurement-Based Probabilistic Power Flow Using a Basis Constrained Graph Convolutional Network with Few-Shot Node Adaptation

**DOI:** 10.3390/s26175662

**Published:** 2026-09-06

**Authors:** Jinbao Wang, Jun Liu, Haobo Zhang, Bairen An, Chencong Zhao

**Affiliations:** 1 School of Automation and Information Engineering, Xi’an University of Technology, Xi’an 710048, China; jbwang@stu.xaut.edu.cn (J.W.); haobozhang@stu.xaut.edu.cn (H.Z.); anbairen@stu.xaut.edu.cn (B.A.); 2 School of Artificial Intelligence and Big Data, Xi’an University, Xi’an 710065, China; zhaochencong@xawl.edu.cn

**Keywords:** probabilistic power flow, Graph Convolutional Network, few-shot adaptation, topology expansion, hybrid orthonormal basis, photovoltaic uncertainty

## Abstract

Probabilistic power flow quantifies voltage and phase angle uncertainty under variable photovoltaic generation, but repeated AC Monte Carlo simulation is costly. A local topology change also modifies the electrical operator and state dimension when only a small target data set is available. We propose a Basis Constrained Graph Convolutional Network (BCGCN) for few-shot adaptation after local bus additions in small-scale grids. BCGCN predicts nonlinear residuals around a first-order solution using graph Laplacian and Proper Orthogonal Decomposition modes. It transfers source coordinates; adapts only the new bus rows, rotation, readouts, and correction gate; and freezes the backbone. The experimental results indicate that BCGCN leads all four reported errors on the IEEE 14 and IEEE 57 expansions. IEEE 118 and Polish 2746 establish the scale boundary. BCGCN wins only 9 of 64 IEEE 118 error cells and none on Polish 2746, while retaining compact updates. The paired IEEE 118 PV study shows that target pilots reduce zero-shot error and residual correction removes most high variability linearization error. BCGCN is therefore effective for local few-shot adaptation in small grids but not an accuracy-preserving adapter for large networks.

## 1. Introduction

The increasing penetration of distributed PV generation makes grid operating states strongly uncertain and raises the need for risk-aware quantities beyond a single deterministic power-flow solution. PPF propagates uncertain injections through the AC power-flow equations to obtain distributions of nodal states and branch flows, supporting weak-link identification and operating-margin assessment [1]. Classical MCS is accurate but computationally expensive [2,3], whereas analytical and point-estimation methods are faster but can lose accuracy under strong nonlinearities [4,5,6]. Measurement-driven surrogates therefore offer a practical route to repeated PPF queries. In this paper, the uncertain PV injections are constructed from historical generation measurements reported for the Hong Kong University of Science and Technology [7]; the supervised AC states and quantiles are generated offline by AC-MCS. The resulting model is a fast PPF surrogate with checkpoint-based few-shot adaptation.

Existing data-driven power-flow surrogates can be broadly divided into Euclidean and graph-based models. DNNs and CNNs provide strong nonlinear fitting on a fixed input layout [8,9], but their representations do not naturally follow changes in graph size or connectivity. GNNs and GCNs instead encode network structure through message passing or graph filters [10,11,12]. Recent work has further introduced physics-guided losses and layers, topology-aware graph operators, scalable graph architectures, and attention or Transformer mechanisms [13,14,15,16,17]. These advances improve physical consistency and cross-operating-condition generalization, but topology expansion remains difficult because the node set and the graph-dependent representation change simultaneously.

Adaptation to a changed topology is commonly handled through full or partial gradient fine-tuning, transfer learning, meta-learned initialization, or topology-conditioned parameter generation [18,19,20]. These approaches are flexible, but they generally adapt or regenerate graph-coupled network weights. They do not by themselves preserve an explicit correspondence between source-network modes and the modes of a larger graph. This distinction motivates both the structured basis transfer proposed here and the inclusion of conventional GNN, transfer-learning, and meta-learning baselines in the experiments. Three connected gaps remain.

First, data-driven surrogates remain structurally fragile under local topology changes and have limited few-shot transfer capability [21]. Maintenance, switching, and grid expansion alter both the graph and its input–output mapping [22,23]. Without a targeted transfer mechanism, accuracy recovery commonly requires many new power-flow samples and broad retraining [14,18].

Second, accuracy-oriented models often couple their parameter spaces deeply to a particular graph [12,13]. Although graph Laplacians and adjacency relations provide useful priors, the learned representation can still become tied to source-graph features [15,24]. When new buses are added, this implicit coupling offers no direct rule for extending the learned coordinates, and transfer can reduce to empirical weight fine-tuning without a geometry-preserving interface [16,25].

Finally, the way physical priors are imposed creates an accuracy–transferability trade-off that is rarely isolated experimentally. A linearized baseline or a topology-derived basis narrows the free residual space and may improve adaptation, but it can also reduce source-topology fitting freedom [20,26]. Distinguishing a genuine representational limitation from the effects of basis dimension, nonlinear correction, or optimization therefore requires controlled component ablations under both source and expanded topologies.

These gaps call for a surrogate that supports structured few-shot transfer while making the accuracy and transferability trade-off measurable. We therefore propose a Basis Constrained Graph Convolutional Network (BCGCN) for PPF. BCGCN is designed for small-scale grids with local bus additions, where a compact modal interface can represent the target correction without updating the full predictor. First-order linearized power flow supplies the physical baseline. The residual representation combines topology-weighted Laplacian modes, which encode grid geometry, with POD modes, which capture recurrent nonlinear residual variation that topology alone does not describe. A ChebConv backbone predicts modal residual coefficients. After node addition, zero-padded source modes serve as anchors, trainable new bus rows extend the basis, a differentiable QR retraction restores rectangular orthonormality, and a Cayley map applies a near identity rotation within the modal subspace. Unlike full gradient, meta initialized, or hypernetwork style adaptation of the backbone, this design exposes a small geometrically defined parameter block for updating. The IEEE 118 and Polish 2746 studies test the point at which this small-scale design no longer preserves the accuracy lead. To address the three research gaps, this paper makes the following contributions.

1. We propose a modular surrogate that separates the first-order linearized response, the orthonormal hybrid basis, the ChebConv backbone, and the coefficient rotation layer. This separation isolates topology-dependent operators from network weights and provides explicit interfaces for targeted fine-tuning.
2. We introduce an anchored basis transfer mechanism with exact orthogonality control for few-shot adaptation after node addition. Zero padding preserves the original modal coordinates as initialization anchors but is not treated as an eigendecomposition of the expanded network. A differentiable QR retraction maps the raw expanded basis to the Stiefel manifold, after which a Cayley rotation adjusts its modal coefficients. Fine-tuning is restricted to new bus rows, the rotation generator, the readout heads, and a scalar correction gate, while the ChebConv backbone remains frozen.
3. We establish the operating range of the compact adapter through a common six-method evaluation on IEEE 14, IEEE 57, IEEE 118, and Polish 2746 bus expansions. IEEE 14 and IEEE 57 test the intended small-scale setting. The IEEE 118 and Polish systems measure the loss of accuracy as the topology and residual field grow. Component ablations, update cost and runtime measurements, and an IEEE 118 PV variability study identify the role of the constrained interface and its current limit.

The remainder of this paper first formulates the PPF task in Section 2, and it then presents the BCGCN architecture, hybrid basis, and transfer mechanism in Section 3. Section 4 reports the IEEE 14-, 57-, 118-, and Polish 2746-bus comparisons, ablations, stress tests, and runtime results. Section 5 summarizes the findings and their limits.

## 2. Problem Statement

For a power grid, PPF can be formulated as a mapping from uncertain power injections to nodal voltage magnitudes and phase angles:

(1) $$\tilde{s}=f\left(u\right),$$

where u=[P;Q] denotes the uncertain net-injection vector at each bus, $\tilde{s}$ represents the vector of nodal voltage magnitudes and phase angles, and *f* is the nonlinear power-flow operator of the grid. Because the input *u* is stochastic, the resulting outputs also exhibit uncertainty.

To improve the generality of the proposed framework, we adopt a quantile-based representation that remains independent of the underlying probability distribution model. Specifically, we characterize both the input and output uncertainties through quantile functions:

(2) $$Q_{t}:=\left\{P_{t}\in R^{q}\;|\;P_{i,t}=Q_{i,t}\left(q_{j}\right)\;\mathrm{for}\;\mathrm{some}\;j\in \left\{1,\ldots ,L\right\},\;\forall \;i=1,\ldots ,q\right\}$$

where $Q_{i,t}\left(\cdot \right)$ denotes the quantile function of the uncertain power injection at bus *i* and time *t*:

(3) $$Q_{i,t}\left(q_{j}\right)=\inf\left\{p\in R\;|\;F_{i,t}\left(p\right)\geq \tau_{j}\right\}$$

The quantile levels satisfy the standard ordering condition

(4) $$0<\tau_{1}<\tau_{2}<\cdots <\tau_{L}<1.$$

However, when the power grid expands, particularly through the addition of new buses, the underlying mapping function *f* changes accordingly. Existing data-driven methods typically learn this mapping from historical or simulated samples and therefore struggle to adapt to previously unseen topologies. As a result, improving the generalization capability of data-driven PPF models under unknown-topology and few-shot scenarios remains a critical challenge.

To address this issue, we propose a BCGCN. First, we use historical grid data to construct a low-dimensional hybrid basis that combines structural and empirical residual modes. BCGCN then constructs a graph representation in which nodal voltages (V,θ) and injected powers (P,Q) serve as node features. During forward propagation, the model predicts residuals relative to a closed-form linearized baseline and projects their principal component onto the hybrid subspace. A gated correction represents only the orthogonal complement and is penalized during training. The proposed framework addresses three challenges: (1) deriving the linearized baseline and spectral basis from the power-flow equations and network topology; (2) designing graph convolution consistent with the physical coupling structure; and (3) adapting the model to expanded networks without retraining the GNN backbone.

## 3. Proposed Basis Constrained Graph Convolutional Network

**Figure 1:**
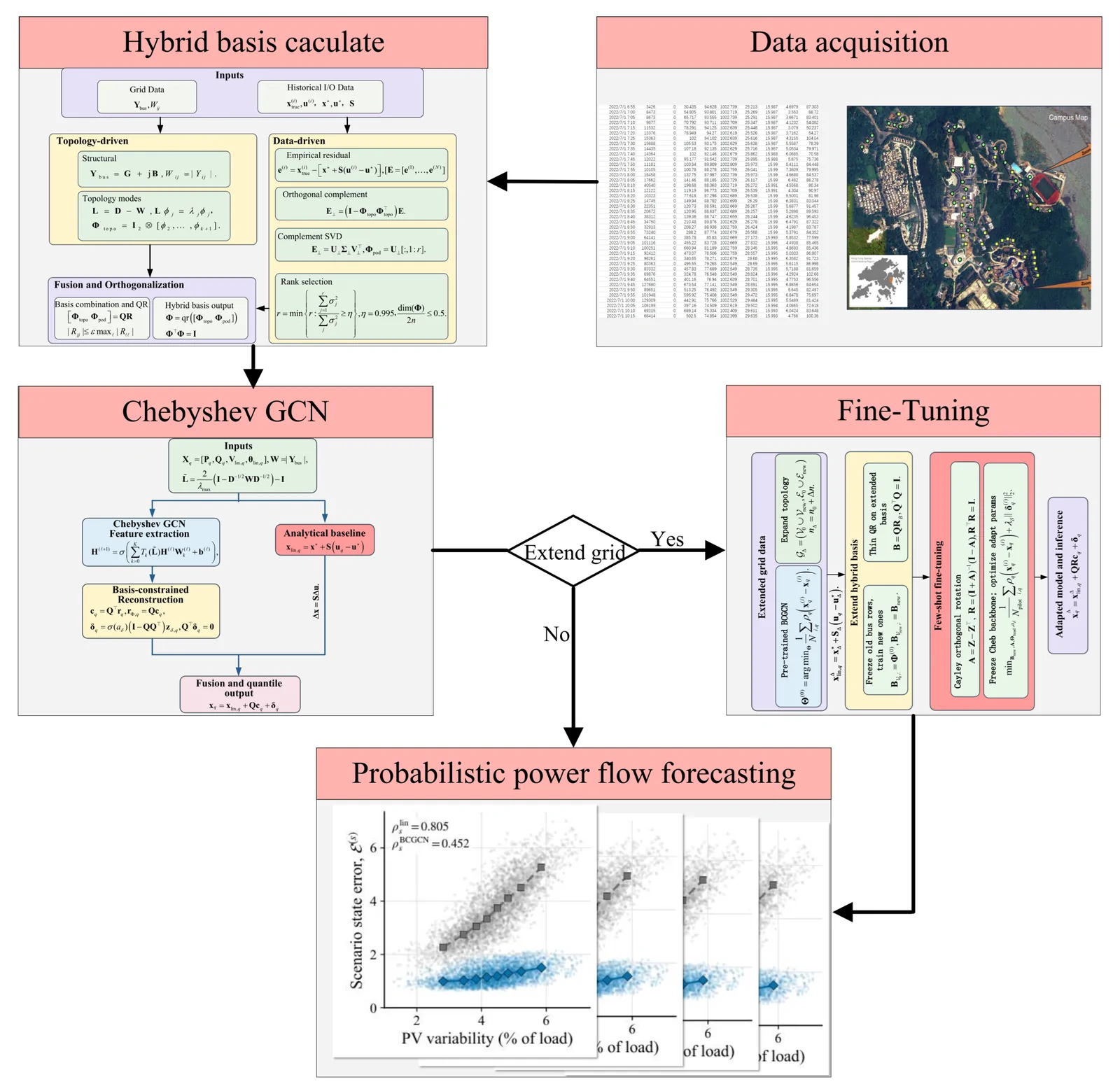
BCGCN workflow for fixed-topology prediction and few-shot topology adaptation; only topology-interface parameters update after expansion.

**Figure 2:**
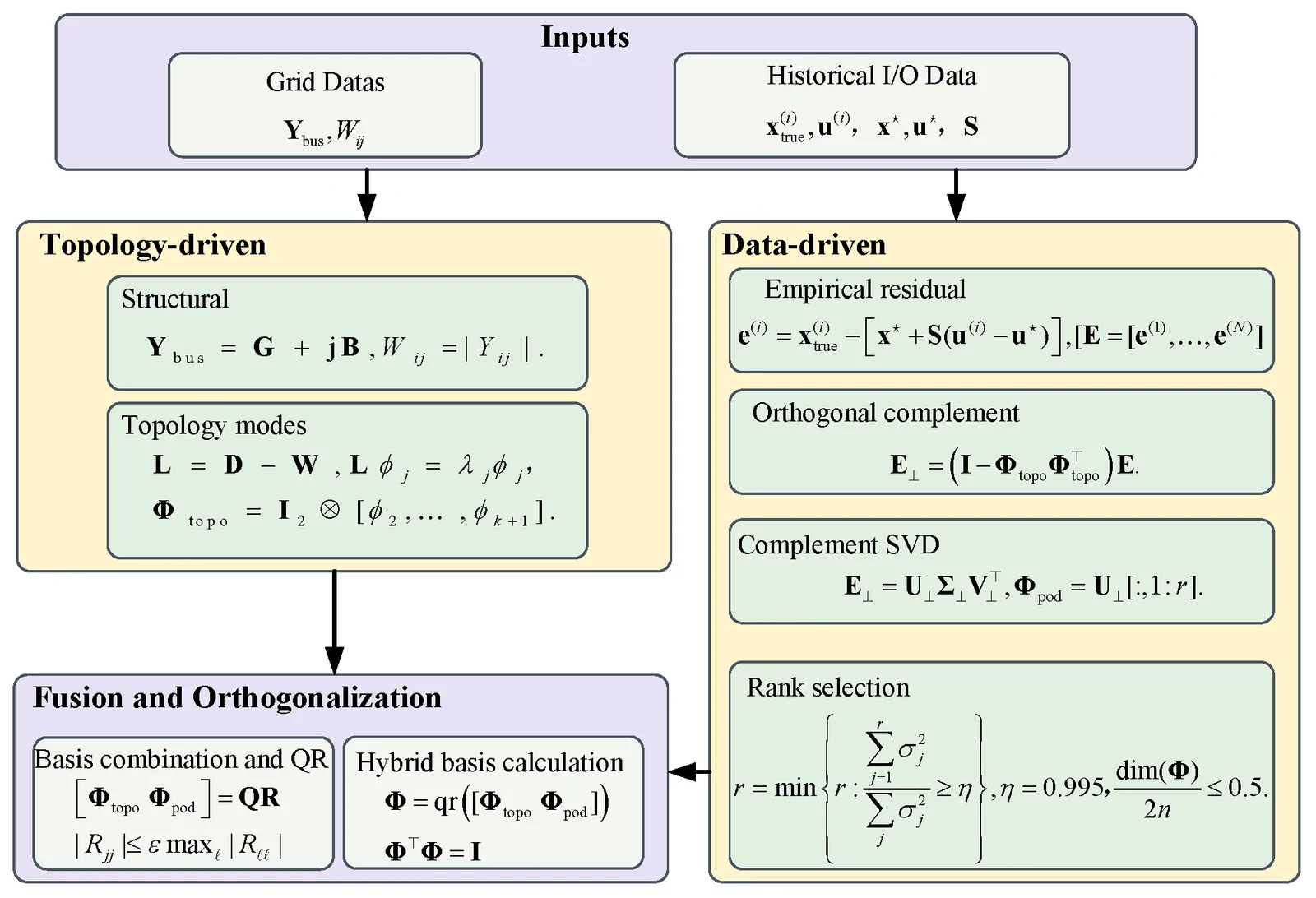
Hybrid basis from admittance-weighted Laplacian modes and POD residual modes; thin QR yields the orthonormal basis Φ.

**Figure 3:**
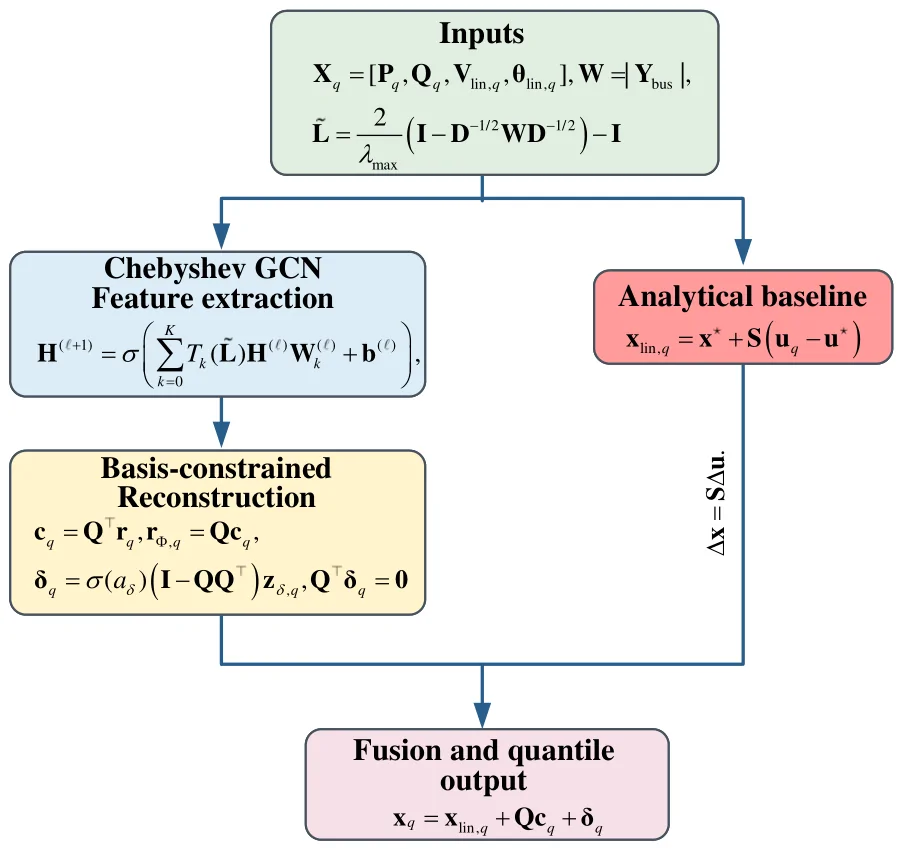
Fixed-topology BCGCN: Chebyshev residual corrections in Φ and gated $\Phi^{⊥}$ augment $x_{\mathrm{lin}}$.

**Figure 4:**
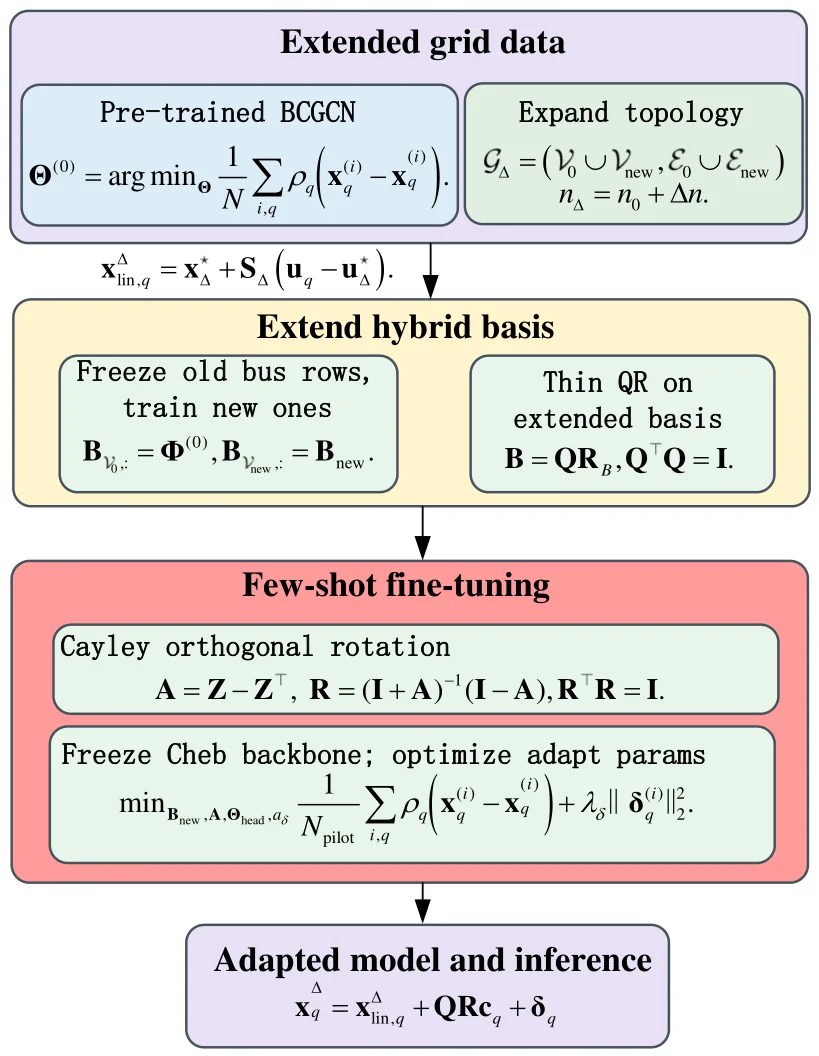
Few-shot adaptation after adding Δn buses. Only new-bus basis rows, Cayley rotation, readouts, and the correction gate update; thin QR preserves orthonormality.

BCGCN separates the power-flow surrogate into an analytical baseline, a basis constrained nonlinear residual, and a gated correction outside the basis subspace. Figure 1 shows how these components connect during source-topology training and how the same checkpoint enters the topology-adaptation path. The hybrid-basis construction, fixed-topology residual predictor, and constrained fine-tuning operation are detailed in Figure 2, Figure 3 and Figure 4. A graph-size-independent Chebyshev backbone is shared by both operating modes; only the interface between its latent representation and the expanded output space is adapted after new buses are introduced.

### 3.1. Linearization Baseline and Hybrid Basis

Building on the nonlinear AC power-flow model introduced in (1), we define the power-mismatch mapping as $f_{\mathrm{AC}}\left(x,u\right)=s_{\mathrm{calc}}\left(x\right)-u=0$, where $x=\left[V;\theta \right]\in R^{2n}$ and $u=\left[P;Q\right]\in R^{2n}$ contains net nodal injections. Writing $Y_{ij}=G_{ij}+jB_{ij}$ and $\theta_{ij}=\theta_{i}-\theta_{j}$, the calculated injections are

(5) $$\begin{array}{cc} P_{i} & =V_{i}\sum_{j=1}^{n}V_{j}\left(G_{ij}\cos\theta_{ij}+B_{ij}\sin\theta_{ij}\right),\\ Q_{i} & =V_{i}\sum_{j=1}^{n}V_{j}\left(G_{ij}\sin\theta_{ij}-B_{ij}\cos\theta_{ij}\right). \end{array}$$

We obtain the nominal point $\left(x^{⋆},u^{⋆}\right)$ from a converged AC power flow and linearize the mismatch equations at that point:

(6) $$J_{x}\;\Delta x+J_{u}\;\Delta u=0,\;J_{x}=\frac{\partial f_{\mathrm{AC}}}{\partial x}{|}_{⋆},\;J_{u}=\frac{\partial f_{\mathrm{AC}}}{\partial u}{|}_{⋆}=-I,$$

where $\Delta x=x-x^{⋆}$ and $\Delta u=u-u^{⋆}$. In the state ordering used by the implementation, the full polar-form Jacobian is

(7) $$J={\left[\begin{array}{cc} \partial P/\partial V & \partial P/\partial \theta \\ \partial Q/\partial V & \partial Q/\partial \theta \end{array}\right]}_{\left(x^{⋆},u^{⋆}\right)}.$$

For i≠j, its entries are

(8) $$\begin{array}{cccc} \frac{\partial P_{i}}{\partial V_{j}}\; & =V_{i}\left(G_{ij}\cos\theta_{ij}+B_{ij}\sin\theta_{ij}\right), & \frac{\partial P_{i}}{\partial \theta_{j}}\; & =V_{i}V_{j}\left(G_{ij}\sin\theta_{ij}-B_{ij}\cos\theta_{ij}\right),\\ \frac{\partial Q_{i}}{\partial V_{j}}\; & =V_{i}\left(G_{ij}\sin\theta_{ij}-B_{ij}\cos\theta_{ij}\right), & \frac{\partial Q_{i}}{\partial \theta_{j}}\; & =-V_{i}V_{j}\left(G_{ij}\cos\theta_{ij}+B_{ij}\sin\theta_{ij}\right), \end{array}$$

and the diagonal entries are

(9) $$\begin{array}{cccc} \frac{\partial P_{i}}{\partial V_{i}} & =\frac{P_{i}}{V_{i}}+G_{ii}V_{i}, & \frac{\partial P_{i}}{\partial \theta_{i}} & =-Q_{i}-B_{ii}V_{i}^{2},\\ \frac{\partial Q_{i}}{\partial V_{i}} & =\frac{Q_{i}}{V_{i}}-B_{ii}V_{i}, & \frac{\partial Q_{i}}{\partial \theta_{i}} & =P_{i}-G_{ii}V_{i}^{2}. \end{array}$$

All quantities in (8) and (9) are evaluated at the nominal point when forming the linear model.

The complete Jacobian in (7) is not inverted directly because the slack and PV controls fix part of the state. Let N denote the non-slack buses and P the PQ buses. The free state and injection perturbations are

(10) $$\Delta x_{r}=\left[\begin{array}{c} \Delta V_{P}\\ \Delta \theta_{N} \end{array}\right],\;\Delta u_{r}=\left[\begin{array}{c} \Delta P_{N}\\ \Delta Q_{P} \end{array}\right].$$

Accordingly, the implementation constructs the reduced Jacobian

(11) $$J_{r}={\left[\begin{array}{cc} \partial P_{N}/\partial V_{P} & \partial P_{N}/\partial \theta_{N}\\ \partial Q_{P}/\partial V_{P} & \partial Q_{P}/\partial \theta_{N} \end{array}\right]}_{\left(x^{⋆},u^{⋆}\right)},\;\Delta x_{r}=J_{r}^{-1}\Delta u_{r}.$$

Let $C_{x}$ and $C_{u}$ select the components in (10) from the full state and injection vectors, respectively. The full-space sensitivity matrix stored by the implementation is    

(12) $$S=C_{x}^{⊤}J_{r}^{-1}C_{u}\in R^{2n\times 2n},$$

and the analytical baseline is

(13) $$x_{\mathrm{lin}}\left(u\right)=x^{⋆}+S\left(u-u^{⋆}\right).$$

This derivation assumes a balanced steady state; a fixed admittance matrix and bus-type assignment during each linearization; fixed transformer taps, shunts, and voltage-control modes; a nonsingular $J_{r}$; and perturbations sufficiently close to the nominal point. The slack-bus voltage magnitude and angle and the PV-bus voltage magnitudes remain fixed. When the topology expands, the nominal AC solution, reduced Jacobian, sensitivity matrix, and graph Laplacian are recomputed for the expanded network rather than transferred from the original topology.

To provide a transferable structural prior, we first construct a topology-dependent basis from the original graph. Its coordinates can be transferred as anchors when buses are added, but the effective expanded basis requires the retraction described later. Using admittance magnitudes as edge weights, we define the weighted Laplacian

(14) $${\tilde{W}}_{ij}=\left\{\begin{array}{cc} |Y_{ij}|, & i\neq j\\ 0, & i=j \end{array}\right.,\;W=\frac{1}{2}\left(\tilde{W}+{\tilde{W}}^{⊤}\right),\;D=\mathrm{diag}\left(W1\right),\;L=D-W,$$

where *L* is symmetric and positive semidefinite. Its eigendecomposition,

(15) $$L\varphi_{i}=\lambda_{i}\varphi_{i},$$

with $0=\lambda_{1}<\lambda_{2}\leq \cdots$, yields the graph Fourier modes. Each eigenvector $\varphi_{i}$ represents a spatial variation pattern across buses, while the corresponding eigenvalue $\lambda_{i}$ quantifies its smoothness over electrically adjacent nodes. Excluding the trivial uniform mode $\varphi_{1}$, we collect the next *k* lowest-frequency modes into

(16) $$\Phi_{\mathrm{node}}=\left[\varphi_{2},\ldots ,\varphi_{k+1}\right]\in R^{n\times k}.$$

Since the system state $x=\left[v_{m};\;v_{a}\right]\in R^{2n}$ contains both voltage magnitudes and phase angles defined on the same graph, the basis acts independently on each component through a Kronecker product:

(17) $$\Phi_{\mathrm{topo}}\;=\;I_{2}⊗\Phi_{\mathrm{node}}\;\in \;R^{2n\times 2k}.$$

When buses are added, zero padding preserves the modal coordinates of the original buses and therefore provides a deterministic transfer anchor. The zero-padded columns are not, in general, eigenvectors of the expanded-network Laplacian because the new branches change both node degrees and electrical couplings. Section 3.3 therefore treats zero padding only as an initialization and restores orthonormality after the new-bus rows are inserted.

Although the topological basis encodes physically meaningful structural priors, it cannot fully capture nonlinear operational behaviors. We therefore complement it with a data-driven basis derived through Proper Orthogonal Decomposition (POD) using historical input–output data ${\left\{\left(u^{\left(i\right)},x_{\mathrm{true}}^{\left(i\right)}\right)\right\}}_{i=1}^{N}$, where each input $u^{\left(i\right)}$ denotes a nodal injection profile and $x_{\mathrm{true}}^{\left(i\right)}$ the corresponding AC power-flow solution. For each sample pair, we compute the linearization residual

(18) $$e^{\left(i\right)}\;=\;x_{\mathrm{true}}^{\left(i\right)}-x_{\mathrm{lin}}^{\left(i\right)},\;x_{\mathrm{lin}}^{\left(i\right)}=x^{⋆}+S\;\left(u^{\left(i\right)}-u^{⋆}\right),$$

which isolates the nonlinear component not captured by the analytical baseline. Stacking these residuals column-wise forms the snapshot matrix

(19) $$E=\left[e^{\left(1\right)},\ldots ,e^{\left(N\right)}\right]\in R^{2n\times N}.$$

The implementation first removes the part already represented by the topological basis and then applies SVD:

(20) $$E_{⊥}=\left(I-\Phi_{\mathrm{topo}}\Phi_{\mathrm{topo}}^{⊤}\right)E,\;E_{⊥}=U_{⊥}\Sigma_{⊥}V_{⊥}^{⊤},\;\Phi_{\mathrm{pod}}^{⊥}=U_{⊥,:,1:r}.$$

When *r* is not prescribed, the code selects the smallest value whose combined captured energy reaches the threshold η, subject to the numerical rank and the cap m/(2n)≤β:

(21) $$r=\min\;\left\{j:\frac{∥\Phi_{\mathrm{topo}}^{⊤}{E∥}_{F}^{2}+\sum_{s=1}^{j}\sigma_{⊥,s}^{2}}{{∥E∥}_{F}^{2}}\geq \eta \right\},\;2k+r\leq \left\lfloor 2n\beta \right\rfloor .$$

If the threshold is infeasible under these constraints, the maximum admissible rank is used. Finally, a reduced QR factorization removes numerical redundancy from the concatenated modes:

(22) $$\left[\Phi ,R_{\Phi }\right]=\mathrm{qr}\;\left([\Phi_{\mathrm{topo}}|\Phi_{\mathrm{pod}}^{⊥}]\right),\;\Phi^{⊤}\Phi =I_{m},\;m\leq 2k+r,$$

This procedure is implemented with SVD and Householder QR rather than sequential classical Gram–Schmidt. SVD supplies mutually orthogonal directions in the complement of $\Phi_{\mathrm{topo}}$, while Householder reflectors reduce the accumulation of roundoff error when the topology and POD blocks are concatenated. The numerical rank of $E_{⊥}$ is estimated by retaining singular values satisfying

(23) $$\sigma_{⊥,j}>\varepsilon_{\mathrm{rank}}\max\left\{\sigma_{⊥,1},1\right\},\;\varepsilon_{\mathrm{rank}}=10^{-10}.$$

After the reduced QR factorization, a column is retained only if

(24) $$|{\left(R_{\Phi }\right)}_{jj}|>\varepsilon_{\mathrm{qr}}\max_{l}\left|{\left(R_{\Phi }\right)}_{ll}\right|,\;\varepsilon_{\mathrm{qr}}=10^{-10}.$$

The default implementation uses η=0.995 and β=0.5 in (21); a prescribed POD rank is still capped by the numerical rank and the same basis-dimension limit. These steps remove nearly dependent directions and keep the normalized Gram error $∥\Phi^{⊤}\Phi -I_{m}{∥}_{F}/m$ at numerical precision. Figure 2 illustrates the complete construction process.

### 3.2. Graph Convolutional Residual Predictor

At runtime, the stored hybrid basis is also passed through the signed thin-QR retraction in (41). Because the source basis is already orthonormal, this step preserves its projector; we therefore retain the notation Φ for original-topology training.

The linearized baseline and hybrid basis jointly define the expected manifold of the nonlinear residual

(25) $$e\left(u\right)=f_{\mathrm{AC}}\left(u\right)-x_{\mathrm{lin}}\left(u\right),$$

but they do not determine how this residual evolves under changing power injections. To model this relationship, we employ a Chebyshev Graph Convolutional Network, as illustrated in Figure 3. Because the network operates on the same admittance graph used to construct $\Phi_{\mathrm{topo}}$, its predictions remain consistent with the underlying electrical coupling structure. The principal residual is subsequently projected onto Φ to enforce a low-rank physical prior, while a parallel correction head represents only the component outside the basis subspace.

#### Notation

Throughout this subsection, *B* denotes the mini-batch size, *L* the number of operating-condition quantiles provided as inputs, *n* the number of buses, *K* the Chebyshev polynomial truncation order, $n_{\mathrm{layers}}$ the number of stacked spectral convolution layers, and $d_{l}$ the hidden width of the *l*-th layer (with $d_{h}:=d_{n_{\mathrm{layers}}}$ denoting the final embedding width).

We construct the layer-0 graph representation by stacking, at each bus and input quantile, the nodal injections $\left(P_{\mathrm{inj}},Q_{\mathrm{inj}}\right)$ and the corresponding linearized state $\left(v_{m,\mathrm{lin}},v_{a,\mathrm{lin}}\right)$ from (13). Concatenating along the feature dimension yields

(26) $$X\in R^{B\times n\times 4L},$$

and we set $H^{\left(0\right)}=X$. The output-state quantiles are linearly interpolated to the same *L*-level grid, and phase angles are converted to radians before training. Each feature channel is standardized using statistics fitted on the original-topology training set.

To propagate information according to the physical coupling structure, we reuse the weighted adjacency matrix *W* employed in the construction of $\Phi_{\mathrm{topo}}$ and define the normalized Laplacian

(27) $$L_{\mathrm{sym}}=I-D^{-1/2}WD^{-1/2},\;\tilde{L}=\frac{2}{\lambda_{\max}\left(L_{\mathrm{sym}}\right)}\;L_{\mathrm{sym}}-I.$$

This rescaling confines the spectrum $\sigma (\tilde{L})$ to the interval [−1,1]. The operator $\tilde{L}$ computes weighted differences between electrically adjacent buses, ensuring that both the graph convolution operator and the spectral basis originate from the same physical topology.

The hidden representation is updated through $n_{\mathrm{layers}}$ stacked Chebyshev spectral convolution layers. Each layer applies a *K*-term polynomial filter of maximum degree K−1, generated through the standard Chebyshev recurrence

(28) $$T_{0}\left(\tilde{L}\right)=I,\;T_{1}\left(\tilde{L}\right)=\tilde{L},\;T_{k}\left(\tilde{L}\right)=2\tilde{L}\;T_{k-1}\left(\tilde{L}\right)-T_{k-2}\left(\tilde{L}\right).$$

The update rule for the *l*-th layer is

(29) $$H^{\left(l\right)}\;=\;\mathrm{ReLU}\;\left(\sum_{k=0}^{K-1}T_{k}\left(\tilde{L}\right)\;H^{\left(l-1\right)}\;W_{k}^{\left(l\right)}\;+\;b^{\left(l\right)}\right),$$

where $W_{k}^{\left(l\right)}\in R^{d_{l-1}\times d_{l}}$ denotes the learnable filter coefficients of order *k* at layer *l*, and $b^{\left(l\right)}\in R^{d_{l}}$ is the corresponding bias. After $n_{\mathrm{layers}}$ such convolutions, the hidden embedding becomes

(30) $$H\in R^{B\times n\times d_{h}}.$$

This formulation provides two critical advantages. First, because $T_{k}\left(\tilde{L}\right)$ is a degree-*k* polynomial of the Laplacian, each layer aggregates information within a localized (K−1)-hop electrical neighborhood. Second, the learnable weights operate exclusively along the feature dimension and therefore remain independent of the number of buses *n*. Consequently, the same trained backbone can generalize across networks of different sizes, which forms the basis for the topology-adaptive fine-tuning strategy described later.

Two parallel readout heads produce the basis-branch and correction-branch residuals; each head outputs both voltage-magnitude and phase-angle channels at all *L* quantiles. The projected branch produces

(31) $$x_{\mathrm{res}}=\mathrm{Reshape}\left({\mathrm{Head}}_{\mathrm{proj}}\left(H\right)\right)\in R^{BL\times 2n},$$

which is projected onto the hybrid basis:

(32) $$c\;=\;x_{\mathrm{res}}\;\Phi \;\in \;R^{BL\times m}.$$

Since $\Phi_{\mathrm{topo}}$ consists of orthonormal Laplacian eigenvectors and the hybrid basis is retracted by the reduced QR factorization in (22), it satisfies $\Phi^{⊤}\Phi =I_{m}$ so that *c* is the orthogonal projection of $x_{\mathrm{res}}$ onto span(Φ).

The modal coefficients *c* generate the reconstructed residual $c\;\Phi^{⊤}$, which is constrained to lie within the column space of Φ and therefore inherits both the topological smoothness of $\Phi_{\mathrm{topo}}$ and the dominant empirical directions captured by $\Phi_{\mathrm{pod}}^{⊥}$.

Residual components outside the modal subspace, particularly localized high-frequency deviations, are represented by a controlled orthogonal correction. The second head first produces

(33) $$z_{\delta }=\mathrm{Reshape}\left({\mathrm{Head}}_{\delta }\left(H\right)\right)\in R^{BL\times 2n}.$$

We then remove its component in the hybrid subspace and apply a learnable scalar gate:

(34) $$\delta =\alpha \;z_{\delta }\left(I-\Phi \Phi^{⊤}\right),\;\alpha =\mathrm{sigmoid}\left(a_{\delta }\right).$$

Consequently, δΦ=0, so the correction branch cannot duplicate the basis-projected component. The final prediction is

(35) $$\hat{x}\left(u\right)=x^{⋆}+S\;\Delta u+c\;\Phi^{⊤}+\delta .$$

Here, $x^{⋆}+S\;\Delta u$ provides the first-order response, $c\;\Phi^{⊤}$ is the basis constrained principal residual, and δ is a gated correction in its orthogonal complement. We therefore constrain the principal learned residual while explicitly measuring the energy carried by the complementary branch.

The model is trained with the pinball loss used for quantile regression and a weak correction-energy penalty:

(36) $$\rho_{q}\left(e\right)=\max\left\{qe,\left(q-1\right)e\right\},$$

(37) $$L\left(\gamma \right)=\frac{1}{B\;L\;2n}\sum_{b,\ell ,i}\rho_{q_{\ell }}\;\left(x_{b,\ell ,i}^{\mathrm{true}}-{\hat{x}}_{b,\ell ,i}\right)+\lambda_{\delta }\frac{{∥\delta ∥}_{F}^{2}}{B\;L\;2n},$$

where

(38) $$\gamma \;=\;{\left\{\;W_{k}^{\left(l\right)},\;b^{\left(l\right)}\;\right\}}_{l=1,\;k=0}^{n_{\mathrm{layers}},\;K-1}\;\cup \;\theta_{{\mathrm{Head}}_{\mathrm{proj}}}\;\cup \;\theta_{{\mathrm{Head}}_{\delta }}\;\cup \;\left\{a_{\delta }\right\},$$

and $\theta_{{\mathrm{Head}}_{\mathrm{proj}}},\theta_{{\mathrm{Head}}_{\delta }}$ denote the trainable parameters of the two readout heads.

Because the Chebyshev weights are independent of graph size, the backbone can be reused on an expanded graph after its scaled Laplacian is updated. The following transfer procedure constructs an orthonormal effective basis from the transferred anchors rather than assuming that zero padding yields expanded-network eigenvectors.

### 3.3. Topology-Adaptive Fine-Tuning for Newly Added Nodes

When new buses are integrated through distributed generation or new loads, the system dimension expands from $R^{2n_{0}}$ to $R^{2n}$, where $n=n_{0}+\Delta$, and the underlying admittance graph changes accordingly. Retraining BCGCN from scratch under such conditions is undesirable because (i) operational data from the expanded system is typically scarce, and (ii) the pretrained Chebyshev backbone already encodes transferable electrical neighborhood representations that should be preserved. We therefore propose a lightweight fine-tuning strategy that adapts only the newly introduced degrees of freedom while freezing the pretrained backbone, as illustrated in Figure 4.

Because the Chebyshev weights $\left\{W_{k}^{\left(l\right)}\right\}$ are independent of graph size, they can be reused after recomputing the scaled Laplacian $\tilde{L}$ of the expanded network. Let $\Phi^{\left(0\right)}\in R^{2n_{0}\times m}$ denote the pretrained hybrid basis. We first insert zero rows at the new voltage-magnitude and phase-angle positions:

(39) $$\bar{\Phi }=\mathrm{ZeroPad}\;\left(\Phi^{\left(0\right)},\Delta \right)\in R^{2n\times m}.$$

This operation preserves the original modal coordinates and ${\bar{\Phi }}^{⊤}\bar{\Phi }=I_{m}$, but it does not make the columns eigenvectors of the expanded-network Laplacian. The zero rows are then replaced by trainable parameters    

(40) $$B_{\mathrm{new}}\in R^{2\Delta \times m},\;B_{\mathrm{new}}∼N\left(0,\sigma_{0}^{2}\right),\;\sigma_{0}=0.01,$$

which produces a raw transferred matrix $B\in R^{2n\times m}$. In general, $B^{⊤}B\neq I_{m}$. We therefore apply a differentiable thin QR factorization with a deterministic column-sign correction:

(41) $$\left(Q_{0},R_{\mathrm{qr}}\right)=\mathrm{qr}\left(B\right),\;s=\mathrm{sign}\left(\mathrm{diag}R_{\mathrm{qr}}\right),\;Q=Q_{0}\mathrm{diag}\left(s\right),\;Q^{⊤}Q=I_{m}.$$

The sign vector is treated as constant during differentiation. The effective basis *Q* is recomputed before every projection and reconstruction. The original rows of the raw anchor *B* remain frozen, although their effective coordinates in *Q* are globally renormalized by the retraction.

The expanded nominal point $x_{\mathrm{new}}^{⋆}\in R^{2n}$ is obtained by solving the AC power flow once on the enlarged topology at the new nominal injection $u_{\mathrm{new}}^{⋆}$, and the sensitivity matrix $S_{\mathrm{new}}$ is recomputed from the expanded-network reduced Jacobian using (11) and (12); neither operation involves learning. The expanded-network features reuse the channel-wise normalization statistics fitted during original-topology pretraining; the pilot set is not used to refit them.

The transferred modal coefficients are computed as $c=x_{\mathrm{res}}Q$. To permit small coefficient rotations, we introduce a Cayley layer

(42) $$R={\left(I+A\right)}^{-1}\left(I-A\right)=\left(I-A\right){\left(I+A\right)}^{-1},\;A^{⊤}=-A,\;c^{\prime }=c\;R^{⊤},$$

initialized with A=0, which implies R=I at the start of fine-tuning. We choose this identity-centered parameterization because the transferred modal coordinates require a local adjustment rather than an unconstrained replacement. The Cayley transform guarantees R∈SO(m) without an orthogonality penalty and uses exactly m(m−1)/2 independent parameters. A Householder factor represents a reflection with determinant −1; representing a rotation therefore requires a product whose length and parity must be selected, and a single factor does not provide a continuous identity-centered parameterization. Givens rotations can start from the identity, but they require a prescribed sequence of plane rotations and a long ordered product. Applying QR to a free square matrix would introduce $m^{2}$ parameters and a non-unique scale/sign representation, whereas a matrix-exponential parameterization requires evaluating an exponential at every forward pass. For the present few-shot, near-identity update, the Cayley map therefore gives a direct and non-redundant compromise between exact orthogonality and computational simplicity.

The gradient behavior is also favorable near the initialization. For an admissible perturbation dA in the skew-symmetric generator, differentiating (42) gives

(43) $$dR=-2{\left(I+A\right)}^{-1}\left(dA\right){\left(I+A\right)}^{-1}.$$

At A=0, dR=−2dA, so the mapping has a nonzero first-order response at the identity. Moreover, a real skew-symmetric *A* has purely imaginary eigenvalues, and the singular values of I+A are $\sqrt{1+\omega_{j}^{2}}$. Hence I+A is always nonsingular and ${∥\left(I+A\right)}^{-1}{∥}_{2}\leq 1$, which prevents the linear solve from amplifying gradients through a nearly singular inverse. The implementation evaluates the transform with a differentiable linear solve rather than an explicit matrix inverse. Identity initialization, the fine-tuning learning rate, and validation-based early stopping keep the update in the local regime where the differential in (43) remains informative. The Cayley map excludes rotations with eigenvalue −1; this global coverage limitation is acceptable here because adaptation begins at R=I and only seeks a local alignment of transferred modal coordinates.

During adaptation, we freeze the Chebyshev backbone $\left\{W_{k}^{\left(l\right)},\;b^{\left(l\right)}\right\}$ and the original-bus rows of the raw anchor *B*, and optimize only

(44) $$\theta_{\mathrm{ft}}\;=\;\left\{\;B_{\mathrm{new}},\;A,\;\theta_{{\mathrm{Head}}_{\mathrm{proj}}},\;\theta_{{\mathrm{Head}}_{\delta }},\;a_{\delta }\;\right\}.$$

The final reconstruction formula becomes

(45) $$\hat{x}\left(u\right)\;=\;x_{\mathrm{new}}^{⋆}+S_{\mathrm{new}}\;\Delta u\;+\;c\;R^{⊤}Q^{⊤}\;+\;\delta ,$$

where $\delta =\alpha z_{\delta }\left(I-QQ^{⊤}\right)$ remains orthogonal to the effective transferred basis.

Under this design, the topology-independent components of BCGCN, including the Chebyshev kernels and the raw original-bus anchors, remain fixed. The nominal point, sensitivity matrix, and scaled graph Laplacian are recomputed analytically for the expanded network. The number of trainable parameters during fine-tuning scales as

(46) $$O\left(2\Delta m\;+\;m^{2}\;+\;d_{h}L\right),$$

where the three terms correspond to the new basis rows $B_{\mathrm{new}}$, the Cayley generator *A*, and the two readout heads (with *L* denoting the number of input quantiles), respectively. The QR retraction adds $O(2nm^{2})$ arithmetic per forward pass but introduces no trainable parameters, see Algorithm 1.
**Algorithm 1** BCGCN pretraining and few-shot topology adaptation**Require:** Original graph $G_{0}$, residual snapshots $D_{\mathrm{hist}}$, source quantile data $D_{0}$, and levels ${\left\{q_{\ell }\right\}}_{\ell =1}^{L}$**Require:** Optional expanded graph $G_{\Delta }$ and pilot data $D_{p}$**Ensure:** Pretrained parameters $\gamma_{0}$ and, when Δ>0, adapted parameters $\theta_{\mathrm{ft}}$      1:Compute the analytical baseline using (12) and (13)      2:Construct $\Phi_{\mathrm{topo}}$ using (14)–(17)      3:Construct the hybrid basis Φ using (18)–(22)      4:Build *X* and ${\tilde{L}}_{0}$ using (26) and (27); fit source statistics (μ,σ)      5:Initialize γ in (38); keep Φ fixed and disable the Cayley rotation      6:**repeat**      7:     **for** each source mini-batch **do**      8:           Compute *H* using (29)      9:           Compute $\hat{x}$ using (31)–(35)    10:          Update γ with Adam by minimizing (37)    11:     **end for**    12:**until** the validation pinball loss satisfies the early-stopping criterion    13:Restore the best source checkpoint $\gamma_{0}$    14:**if** Δ>0 **then**    15:  Recompute $\left(x_{\mathrm{new}}^{⋆},u_{\mathrm{new}}^{⋆},S_{\mathrm{new}},{\tilde{L}}_{\mathrm{new}}\right)$ and reuse (μ,σ)    16:     Form the raw transferred basis and its effective basis *Q* using (39)–(41)    17:  Load $\gamma_{0}$; freeze the Chebyshev backbone and raw original-bus anchors; optimize only (44)    18:     **repeat**    19:           **for** each pilot mini-batch **do**    20:             Recompute *Q* using (41) and *H* using (29)    21:             Compute the rotated prediction using (42) and (45), with *Q* in (34)    22:             Update only $\theta_{\mathrm{ft}}$ with Adam by minimizing (37)    23:           **end for**    24:     **until** the pilot-validation pinball loss satisfies the early-stopping criterion    25:     Restore the best adapted checkpoint    26:**end if**    27:**return** $\gamma_{0}$ and, when applicable, $\theta_{\mathrm{ft}}$

## 4. Experimental Evaluation

### 4.1. Experimental Setup

The few-shot evaluation covers IEEE 14, IEEE 57, IEEE 118, and Polish 2746 bus systems. BCGCN is designed for small-scale grids with local additions. The IEEE 14 and IEEE 57 systems test this intended setting, whereas IEEE 118 and Polish 2746 test how the same compact interface changes as the network becomes larger. PV uncertainty is constructed from Hong Kong University of Science and Technology generation measurements [7]. A fixed seed assigns source-topology scenario identifiers to training, validation, and test subsets in the proportions of 8, 1, and 1. Every method is evaluated from its saved checkpoint on the same held-out identifiers. The experiments run with Python 3.8.20 and PyTorch 2.4.1+cu121 on an Intel i9-13900K CPU, 64 GB RAM, and an NVIDIA RTX 4080 SUPER GPU with 16 GB memory.

**Figure 5:**
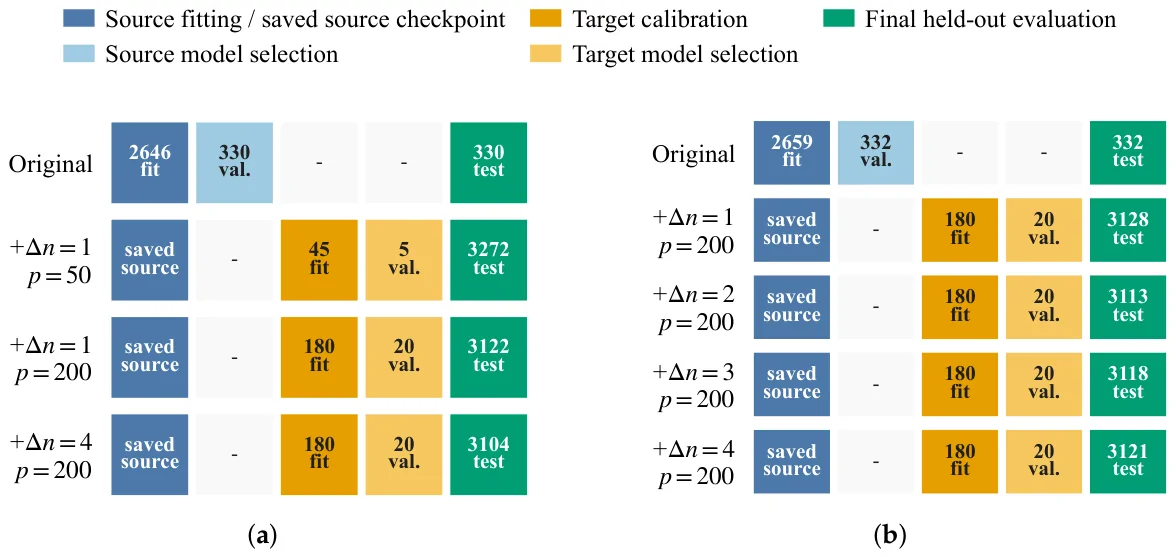
Data roles for (**a**) IEEE 14-bus and (**b**) IEEE 57-bus evaluations. Final test identifiers are disjoint from calibration and model selection and shared by all methods.

Expanded topologies add one to four zero-reactive-power generator buses through tie lines. For the primary four-bus cases, parent/injection sequences are (4,1,3,2)/(8,6,5,5) MW on IEEE 14 and (11,8,2,0)/(20,15,18,10) MW on IEEE 57. We test location sensitivity with two one-parent substitutions per system: (0,1,3,2) and (4,1,3,0) for IEEE 14, and (1,8,2,0) and (11,5,2,0) for IEEE 57. All other settings, including injections, lines, pilots, seeds, and held-out evaluation, are fixed. The IEEE 118 and Polish 2746 sequences are (48,11,91,24)/(40,35,30,25) MW and (2255,1358,238,1232)/(60,50,40,30) MW, respectively; each retains one sequence. IEEE 14 and IEEE 57 use 1 km tie lines with r=0.05Ω/km, x=0.10Ω/km, zero shunt capacitance, and a 1 kA current rating. IEEE 118 uses 8 km tie lines with r=0.06Ω/km, x=0.30Ω/km, and a 0.6 kA current rating. Polish 2746 uses 10 km tie lines with r=0.08Ω/km, x=0.32Ω/km, and a 0.8 kA current rating. An AC solution is recomputed for every specified topology and Monte Carlo draw, and only converged draws are retained. This is an AC-feasibility screen, not an optimal-power-flow, reserve, or capacity-adequacy claim. For every target topology, a seeded permutation selects $N_{\mathrm{pilot}}$ scenarios. Ten percent form the validation set, the remaining 90% are used for adaptation, and all nonpilot scenarios remain held out for testing. The IEEE 118 and Polish 2746 studies each cover the full 4×4 grid of one to four added buses and $N_{\mathrm{pilot}}\in \left\{50,100,150,200\right\}$. Each therefore has 16 target conditions and six-method evaluations per condition. Thus, neither model selection nor fine-tuning observes an expanded topology test case. Figure 5 illustrates the common held-out protocol on the two smaller IEEE systems.

Table 1 reports only the method-defining configuration. Where applicable, the predictors use four hidden layers of width 128. BCGCN uses Chebyshev order K=3. All methods use Adam, a batch size of 64, a source learning rate of $10^{-5}$, an adaptation learning rate of $10^{-6}$, and early stopping with a patience of 30 validation epochs. The comparison covers BCGCN, a conventional GNN [11], DNN [27], CNN [9], TGACN-TL [28], and Meta-MPNN [29].

**Figure 6:**
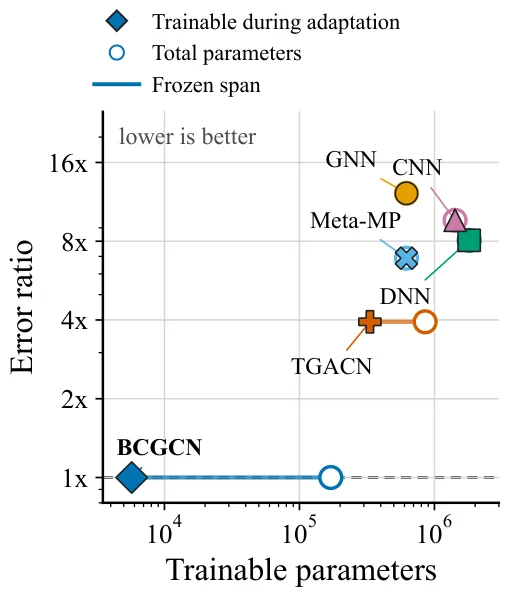
Trainable-to-total parameter span versus the geometric mean of eight error ratios at Δn=4 and $N_{\mathrm{pilot}}=200$; lower left is better.

The six methods cover three transfer mechanisms. DNN and CNN learn a fixed Euclidean input–output layout; GNN and Meta-MPNN follow electrical edges by message passing, with the latter changing the initialization through meta-learning; TGACN-TL combines edge attention with partial fine-tuning. BCGCN instead freezes its graph-size-independent backbone and changes an explicit modal interface and its readouts. On the IEEE comparisons, BCGCN updates 5721 of the 170,585 parameters (3.35%), compared with 38.98% for TGACN-TL and 100% for GNN, DNN, CNN, and Meta-MPNN. The four-bus Polish adapter updates 5859 parameters; its larger target state therefore changes the count but not the localized-update principle. These budgets are fixed before the performance comparisons in Figure 6.

The input and output quantile levels are

$$\begin{array}{cc} \tau^{p}\; & \in \{0.025,0.05,0.15,0.25,0.40,0.50,0.60,0.75,0.85,0.95,0.975\},\\ \tau^{V}\; & \in \{0.05,0.15,0.35,0.50,0.65,0.85,0.95\},\\ \tau^{\theta }\; & \in \{0.05,0.15,0.25,0.35,0.50,0.65,0.75,0.85,0.95\}, \end{array}$$

for uncertain injections, voltage magnitude, and phase angle, respectively.

The raw voltage-magnitude and phase-angle quantiles are interpolated onto the 11-level injection grid before model fitting. Consequently, each bus supplies 44 input features, namely, 11 values for each of $P_{\mathrm{inj}}$, $Q_{\mathrm{inj}}$, $V_{\mathrm{lin}}$, and $\theta_{\mathrm{lin}}$, and each predictor outputs 11 joint voltage-magnitude and phase-angle states. One supervised record is one operating-scenario identifier. Its reference target is formed from 1000 attempted AC Monte Carlo draws after nonconverged draws are removed; the draws are not treated as separate neural records.

All performance indicators used in the experimental subsections are defined here. Let ${\hat{y}}_{i,j}^{z}\left(\tau_{k}\right)$ and $y_{i,j}^{z,*}\left(\tau_{k}\right)$ be the predicted and AC Monte Carlo reference quantiles of state z∈{V,θ}, respectively, and define $e_{i,j}^{z}\left(\tau_{k}\right)={\hat{y}}_{i,j}^{z}\left(\tau_{k}\right)-y_{i,j}^{z,*}\left(\tau_{k}\right)$. For *N* test scenarios, *b* buses, and $L_{z}$ output quantiles,

(47) $$\begin{array}{cc} E_{1}^{z}\; & =\frac{1}{NbL_{z}}\sum_{i=1}^{N}\sum_{j=1}^{b}\sum_{k=1}^{L_{z}}\left|e_{i,j}^{z}\left(\tau_{k}\right)\right|,\\ E_{2}^{z}\; & =\frac{1}{NbL_{z}}\sum_{i=1}^{N}\sum_{j=1}^{b}\sum_{k=1}^{L_{z}}e_{i,j}^{z}{\left(\tau_{k}\right)}^{2},\\ M_{\tau }^{z}\; & =\frac{1}{Nb}\sum_{i=1}^{N}\sum_{j=1}^{b}\left|e_{i,j}^{z}\left(\tau \right)\right|,\;\tau \in \left\{0.50,0.95\right\}. \end{array}$$

$E_{1}^{z}$ and $E_{2}^{z}$ are equal-weight grid errors, not numerical Wasserstein distances, because the voltage and angle levels are nonuniform. For a weighted discrepancy, use $w_{1}=\left(\tau_{2}-\tau_{1}\right)/2$, $w_{k}=\left(\tau_{k+1}-\tau_{k-1}\right)/2$, and $w_{L_{z}}=\left(\tau_{L_{z}}-\tau_{L_{z}-1}\right)/2$, giving $W_{1}^{z}=\sum_{k}w_{k}\left|e_{i,j}^{z}\left(\tau_{k}\right)\right|$ and ${\left(W_{2}^{z}\right)}^{2}=\sum_{k}w_{k}{\left[e_{i,j}^{z}\left(\tau_{k}\right)\right]}^{2}$. The figures use $E_{0.95}^{z}$ for $M_{0.95}^{z}$; voltage errors are presented per unit and angle errors in degrees.

We assess distributional quality on retained raw AC Monte Carlo draws from the held-out scenarios. For state observation *Y* and unrearranged prediction $\hat{Q}$, CRPS uses 11-level trapezoidal pinball-loss integration,

(48) $$\mathrm{CRPS}\left(\hat{Q},Y\right)\approx 2\;{\mathrm{trapz}}_{\tau \in [0,1]}\rho_{\tau }\;\left(Y-\hat{Q}\left(\tau \right)\right).$$

Constant endpoint extrapolation covers [0,0.025] and [0.975,1]. We report the mean absolute coverage error over 20%, 50%, 70%, 90%, and 95% central intervals, pooled over retained test observations, and the adjacent-level crossing rate before any rearrangement.

Ratios expose whether an advantage is confined to a single output or metric. For method *m* and metric–output pair *r*,

(49) $$R_{m,r}=\frac{E_{m,r}}{E_{\mathrm{BCGCN},r}},\;G_{m,r}\left(a\leftarrow b\right)=\frac{E_{m,r}^{\left(a\right)}}{E_{m,r}^{\left(b\right)}}.$$

$R_{m,r}>1$ favors BCGCN. $G_{m,r}\left(a\leftarrow b\right)<1$ denotes an error reduction from condition *b* to *a*; in topology-growth plots, $G_{m,r}\left(4\leftarrow 1\right)>1$ denotes degradation from one to four added buses. Ablation ratios replace the denominator in (49) by the paired full-model error. Their joint summary is the geometric mean $J_{a}=\sqrt{R_{a,E_{1}^{V}}R_{a,E_{1}^{\theta }}}$ and is used only for visualization, not as a training objective.

The IEEE 118-bus analysis reports scenario-level errors over the full probabilistic state. For scenario *s*, the vector stacks 11 quantile states, each containing the voltage magnitude per unit and the phase angle in radians:

(50) $$E_{\mathrm{lin}}^{\left(s\right)}={∥x_{\mathrm{AC}}^{\left(s\right)}-x_{\mathrm{lin}}^{\left(s\right)}∥}_{2},\;E_{\mathrm{BCGCN}}^{\left(s\right)}={∥x_{\mathrm{AC}}^{\left(s\right)}-{\hat{x}}_{\mathrm{BCGCN}}^{\left(s\right)}∥}_{2}.$$

Two diagnostics accompany these norms:

(51) $$\nu_{\mathrm{PV}}^{\left(s\right)}=\frac{\sum_{i}\;\left(P_{i,0.95}^{\left(s\right)}-P_{i,0.05}^{\left(s\right)}\right)}{\sum_{j}P_{\mathrm{load},j}},\;C^{\left(s\right)}=1-\frac{E_{\mathrm{BCGCN}}^{\left(s\right)}}{\max(E_{\mathrm{lin}}^{\left(s\right)},10^{-12})},\;{\bar{E}}^{\left(s\right)}=\frac{E^{\left(s\right)}}{\sqrt{22n_{s}}}.$$

Here, $\nu_{\mathrm{PV}}^{\left(s\right)}$ is the aggregate 5–95% PV width normalized by total active load, $C^{\left(s\right)}$ is the fraction of linearization error removed by BCGCN, and ${\bar{E}}^{\left(s\right)}$ removes the direct effect of the $22n_{s}$ state dimension.

Runtime is characterized by the median and 95th-percentile latency of 200 CUDA-event repetitions after 30 warm-up passes, with tensors resident on the GPU and explicit synchronization. Throughput is batch size 64 divided by the median batch latency. An adaptation step comprises one in-memory forward pass, loss evaluation, backward pass, and Adam update at batch size 32; its median and 95th percentile use 20 repetitions after five warm-up steps. Multi-run plots show individual matched runs together with the median and interquartile range; distribution panels additionally show 10–90% whiskers where present. Original- and expanded-topology checkpoint comparisons are single deterministic evaluations per stated condition and therefore carry no artificial replicate error bars.

### 4.2. Overall Performance on Original Topologies

**Figure 7:**
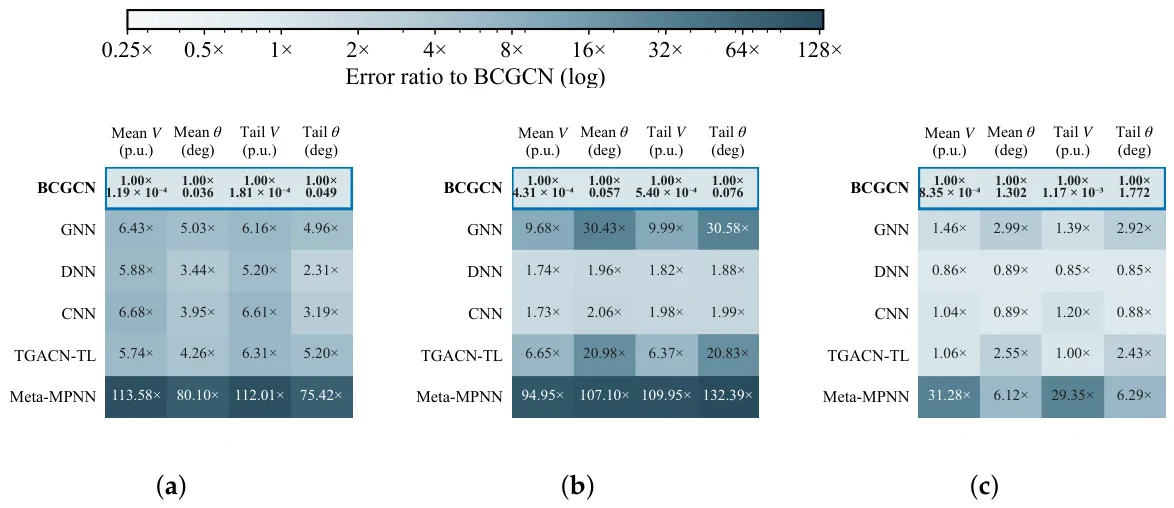
Original-topology errors relative to BCGCN on (**a**) IEEE 14-bus, (**b**) IEEE 57-bus, and (**c**) Polish 2746-bus systems. Columns show the mean and 0.95-quantile voltage and angle errors; BCGCN cells give absolute values. Ratios below 1 denote a baseline with lower error than BCGCN.

BCGCN has the lowest mean and 0.95-quantile errors for both state variables on the IEEE 14-bus and IEEE 57-bus original networks, across all six models, as shown in Figure 7. On the IEEE 14-bus system, $E_{1}^{V}=1.19\times 10^{-4}$ p.u. and $E_{1}^{\theta }=0.0356^{\circ }$. The strongest competing values are $6.82\times 10^{-4}$ p.u. from TGACN-TL and $0.1226^{\circ }$ from DNN, corresponding to 5.74-fold and 3.44-fold larger errors. At the 0.95 quantile, the best baseline remains 5.20 times worse for voltage magnitude and 2.31 times worse for phase angle.

The numerical lead gives the dimensionality reduction a concrete meaning. The analytical term removes the dominant local response around the nominal operating point, so ChebConv fits a residual rather than the full AC map. Laplacian modes organize electrically smooth residuals and POD modes retain recurrent nonlinear directions; their shared coordinates serve both voltage magnitude and phase angle. On IEEE 14-bus, this restricted prediction path still beats every baseline in all four cells by factors of 2.31–6.61; on IEEE 57-bus, it retains 1.73–2.06-fold mean-error and 1.82–1.99-fold tail-error margins. The complementary branch is essential: it captures residual energy outside the retained modes, preventing the modal restriction from converting a lower-dimensional fit into an underfit model. Projection therefore reduces the coefficient space that must be learned while the measured source and tail errors show that the discarded directions are not simply ignored.

The margin narrows on the larger IEEE 57-bus network but does not disappear. BCGCN attains $E_{1}^{V}=4.31\times 10^{-4}$ p.u. and $E_{1}^{\theta }=0.0572^{\circ }$; the best baseline errors are 1.73 and 1.96 times larger. Its 0.95-quantile errors, $5.40\times 10^{-4}$ p.u. and $0.0760^{\circ }$, improve on the strongest competitors by factors of 1.82 and 1.88. Because every baseline cell in the two dominance matrices exceeds parity, the gain is not produced by one unusually favorable output or by averaging away a tail failure.

The baseline pattern clarifies which part of the formulation matters. DNN and CNN must learn the complete nonlinear mapping in a fixed coordinate layout; the former uses global dense mixing and the latter imposes local convolution on bus order, which is not the same as electrical adjacency. GNN and Meta-MPNN follow the electrical graph, but they still predict the complete state directly and have no explicit linear anchor or modal residual split. TGACN-TL adds topology-aware attention, whereas its transfer block is inactive on the unchanged source graph and therefore cannot itself explain a source-topology advantage. BCGCN’s four-metric lead is consequently consistent with the conditioning provided by the analytical-plus-residual decomposition, rather than with graph connectivity alone.

The smaller ratios on IEEE 57 also delimit that interpretation. A larger network introduces longer interaction paths and a higher-dimensional residual field, so a fixed modal budget must summarize a broader set of spatial responses. The correction branch prevents a sharp loss, but the reduced margin indicates that the basis does not make complexity disappear. The fact that the 0.95-quantile ratios remain above 1 is important here: the residual coordinates continue to cover high-quantile behavior rather than merely reducing average error around median operating conditions.

Figure 7c and Table 2 extend the controlled source-topology comparison to the Polish 2746-bus system, using the same 2664/332/332 held-out split for all six saved checkpoints. BCGCN uses only 0.171 million trainable parameters and obtains a Pinball loss of $2.041\times 10^{-3}$, $E_{1}^{V}=8.35\times 10^{-4}$ p.u., and $E_{1}^{\theta }=1.302^{\circ }$. It has lower error than GNN and Meta-MPNN on all four figure metrics, and it is also lower than TGACN-TL in all four cells, although the tail-voltage margin is only 0.14%. The dense DNN and CNN improve Pinball loss by 12.6% and 9.3%, respectively, and DNN is lower on all four errors; this establishes a real source-fit trade-off rather than a universal accuracy lead. Their 182.015 and 181.449 million parameters are nevertheless 1067 and 1064 times the BCGCN count. Thus, at this scale, the constrained representation retains a favorable accuracy–model size compromise and a clear advantage over the conventional graph and meta-learning baselines, while the dense-model advantage remains explicit. This unchanged-topology result establishes only scale and parameter growth; the following Polish expansion analysis tests whether that compromise survives few-shot transfer.

### 4.3. Few-Shot Adaptation to Expanded Topologies

#### 4.3.1. Common Six-Method Comparisons

**Figure 8:**
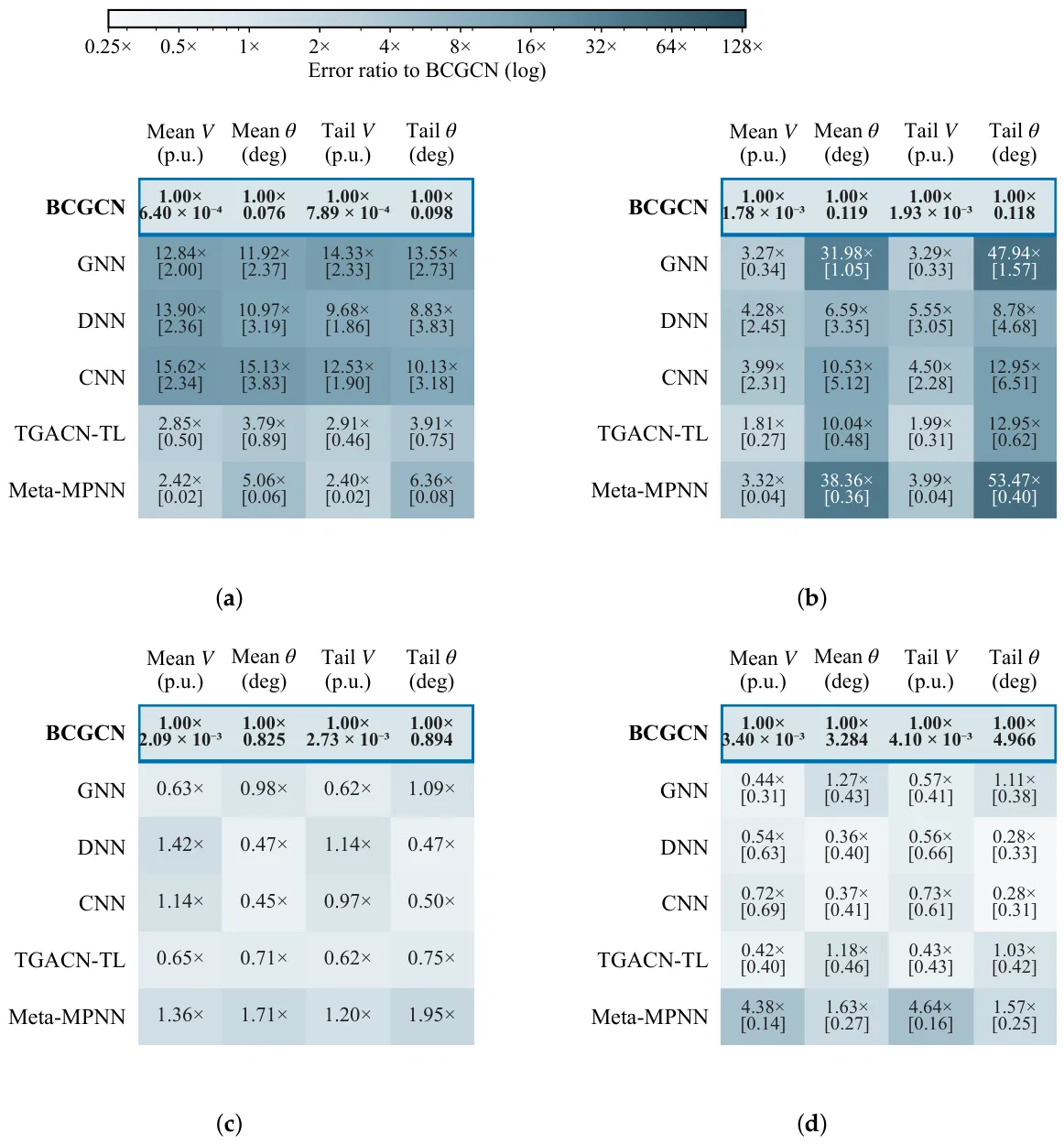
Few-shot errors relative to BCGCN after four added buses with $N_{\mathrm{pilot}}=200$ on the (**a**) IEEE 14, (**b**) IEEE 57, (**c**) IEEE 118, and (**d**) Polish 2746 bus systems. BCGCN cells give the absolute errors. For every baseline, the first line gives its error ratio to BCGCN. In panels (**a**,**b**,**d**), the bracketed second line normalizes that ratio by the corresponding original topology ratio. Ratios below 1 favor the baseline. Panel (**c**) reports direct ratios because IEEE 118 is used here to test few-shot scalability rather than source-topology performance.

The common six-method comparison uses four added buses and 200 pilot scenarios on IEEE 14, IEEE 57, IEEE 118, and Polish 2746 bus systems. Figure 8 compares the same four errors and six saved checkpoints in all four systems. BCGCN remains the best on all four errors in the two small-scale systems shown in panels (a) and (b). On the IEEE 14 bus expansion, it reaches $E_{1}^{V}=6.40\times 10^{-4}$ p.u. and $E_{1}^{\theta }=0.0758^{\circ }$. The best baseline values are 2.42 and 3.79 times larger. Its 0.95 quantile advantage is similarly broad, with 2.40 times lower voltage error and 3.91 times lower phase-angle error.

Node addition changes both the electrical operator and the output coordinates. BCGCN recomputes the nominal AC solution, sensitivity matrix, and graph operator for the target topology, so its first-order term already represents the new branches. In the nonlinear path, zero-padded source modes preserve coordinates on existing buses and trainable new-bus rows add only the missing coordinates. QR retraction converts this extension into an orthonormal effective basis, while the readouts map frozen latent features to the enlarged output. The resulting pilot gradients update the topology interface rather than the entire predictor.

The separation is larger for phase angle on the IEEE 57 bus expansion. BCGCN records $E_{1}^{V}=1.78\times 10^{-3}$ p.u. and $E_{1}^{\theta }=0.1194^{\circ }$. These errors are 1.81 and 6.59 times below the best alternatives. At the 0.95 quantile, the corresponding factors are 1.99 and 8.78. Hence the source-topology advantage survives both a larger output space and a disjoint few-shot adaptation set, rather than merely reflecting a favorable original topology fit.

The IEEE 118 bus results reveal the transition from the intended small-scale regime to the large network boundary. Across its 16 target conditions, BCGCN ranks first in 9 of the 64 error cells. All nine wins are voltage errors with one or two added buses. With one added bus and 200 pilots, it attains the best $E_{1}^{V}=8.92\times 10^{-4}$ p.u. and $E_{0.95}^{V}=1.15\times 10^{-3}$ p.u., but its phase-angle errors remain 1.51 and 1.44 times the best values. With four added buses and 200 pilots, BCGCN records $E_{1}^{V}=2.09\times 10^{-3}$ p.u., $E_{1}^{\theta }=0.825^{\circ }$, $E_{0.95}^{V}=2.73\times 10^{-3}$ p.u., and $E_{0.95}^{\theta }=0.894^{\circ }$. These values are 1.58, 2.21, 1.61, and 2.12 times the respective best baselines in panel (c). It no longer has an accuracy lead, but it updates 5820 parameters while the baselines update 0.333 to 9.041 million. The result shows that localized adaptation remains economical at IEEE 118, while the fixed modal interface no longer covers all voltage and phase angle residual directions.

The Polish 2746 bus expansion gives the stronger large network limit. All 16 Polish target conditions have four node addition levels, four pilot set sizes, and six saved checkpoints per condition. BCGCN does not rank first in any of the 64 error cells. At four added buses and 200 pilots, it records $E_{1}^{V}=3.40\times 10^{-3}$ p.u., $E_{1}^{\theta }=3.284^{\circ }$, $E_{0.95}^{V}=4.10\times 10^{-3}$ p.u., and $E_{0.95}^{\theta }=4.966^{\circ }$. The respective best baseline errors are 0.419, 0.357, 0.434, and 0.277 times these values in Figure 8d. The full resolution dense DNN and CNN models dominate the angle errors, and GNN or TGACN TL dominate the voltage errors. BCGCN nevertheless has lower Pinball loss than GNN, TGACN TL, and Meta MPNN in this condition, with 0.00579 versus 0.00714, 0.00661, and 0.01007. It adapts only 5859 parameters instead of 0.333 to 182.279 million. The compact interface therefore retains an update cost advantage, not an accuracy advantage, at this scale. The IEEE 118 and Polish results make the scope explicit. BCGCN is designed for few-shot local adaptation in small-scale grids, not as a universally superior accuracy model for large networks.

The complete Polish grid also reveals what additional pilot data can and cannot repair. At four added buses, increasing the pilot set from 50 to 200 lowers BCGCN’s mean voltage and angle errors by 13.2% and 8.1%, respectively, but raises its 0.95-quantile voltage error by 3.2%. With 200 pilots, moving from one to four added buses raises its mean voltage, mean angle, tail voltage, and tail angle errors by 27.1%, 14.2%, 60.4%, and 20.0%. These trends are consistent with a fixed modal interface becoming insufficient for the larger target residual, but they do not identify modal rank as the sole cause. They delimit the result precisely: the proposed adapter remains inexpensive to update, whereas the IEEE accuracy lead cannot be extrapolated to the 2746-bus expansion.

The IEEE 57 angle result identifies the part of the representation that transfers most effectively in this regime. At 200 pilots, its mean and tail angle errors are 6.59 and 8.78 times below the best baseline, whereas the voltage margins are 1.81 and 1.99. Phase-angle responses are coupled along electrical paths and are often spatially smooth, so low-frequency weighted-Laplacian modes provide a suitable target coordinate system. Anchoring these coordinates on the original buses limits their drift during calibration. DNN and CNN have no comparable graph coordinate, while full fine-tuning of GNN and Meta-MPNN changes both feature extraction and readout from the same pilot set. TGACN-TL preserves more source parameters, but transfers attention features rather than an explicitly orthonormal source modal space. This controlled comparison is consistent with the proposed transfer mechanism on IEEE 57; it does not establish that topology awareness alone causes the gap.

**Figure 9:**
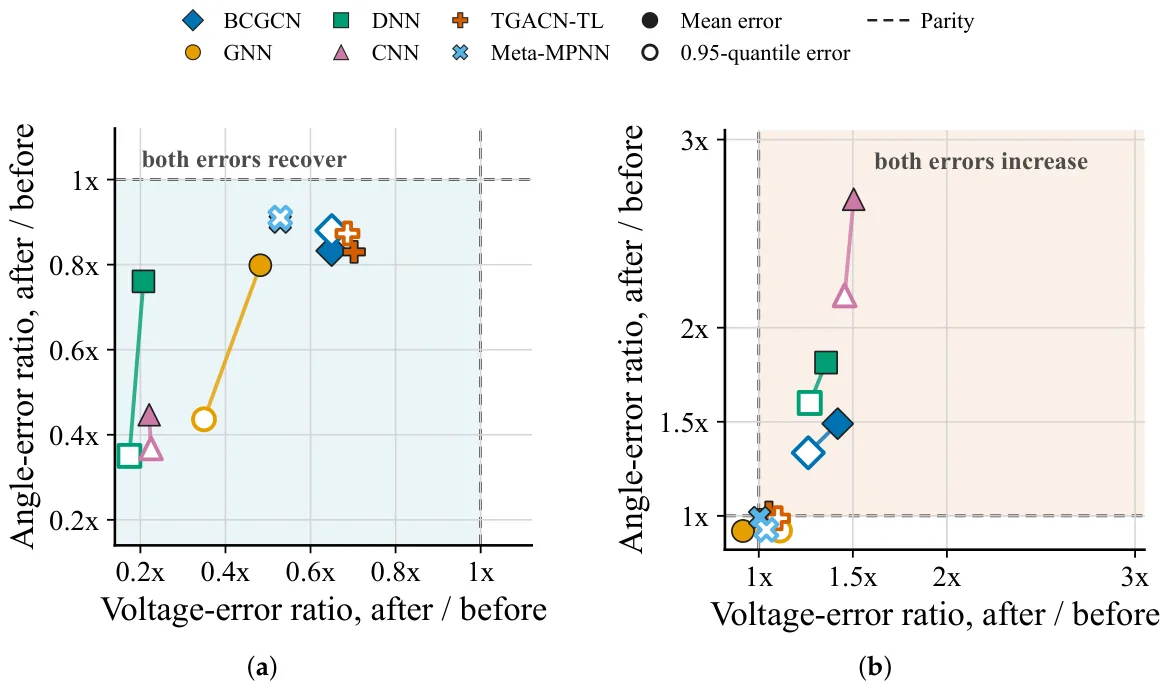
Voltage–angle error ratios after versus before adaptation for (**a**) 50 to 200 pilots at Δn=1 on IEEE 14-bus and (**b**) Δn=1 to 4 at $N_{\mathrm{pilot}}=200$ on IEEE 57-bus. Filled and open markers denote mean and 0.95-quantile errors.

Pilot-set size and expansion severity have distinct effects. With one added bus on IEEE 14-bus, increasing the pilot set from 50 to 200 reduces BCGCN’s mean voltage error from $7.90\times 10^{-4}$ to $5.13\times 10^{-4}$ p.u. and mean phase-angle error from $0.0828^{\circ }$ to $0.0690^{\circ }$; the two tail errors fall by 35.0% and 12.0%. Figure 9a therefore shows a measurable recovery from a small calibration set rather than merely a lower error at one selected pilot size.

The unequal reductions reveal the boundary of that recovery. Voltage improves comparably in its mean and tail, but the phase-angle tail improves less than the mean. This pattern is compatible with early calibration of dominant new-bus modal loadings, which corrects systematic voltage displacement, while rare angle configurations need wider target-distribution coverage. Because the backbone is frozen, more pilots refine the new rows, within-subspace rotation, readouts, and correction gate; they cannot rewrite source feature extraction. The evidence supports interface calibration as the source of the IEEE 14 gain, but the aggregate errors cannot assign that gain to one updated block.

On IEEE 57-bus, the error is controlled rather than invariant as nodes are added. From Δn=1 to Δn=4, BCGCN’s $E_{1}^{V}$, $E_{1}^{\theta }$, $E_{0.95}^{V}$, and $E_{0.95}^{\theta }$ rise by 41.9%, 49.0%, 26.3%, and 33.5%, respectively, yet its four-metric ranking in Figure 8b is unchanged. The 3.35% trainable fraction therefore buys a constrained update with a measured accuracy benefit on this network; it does not make the added buses free.

Each new tie line changes node degrees and electrical coupling, whereas zero padding supplies only an initialization anchor, not the expanded graph’s eigendecomposition. QR maintains mutual orthogonality of the transferred columns but cannot ensure that a fixed rank subspace spans every new residual direction. The complementary branch can repair part of the mismatch. This explains why the IEEE 57 error grows without a rank reversal and why the Polish result is a necessary counterexample. More target complexity can exhaust the same interface. Figure 6 reflects parameter localization. The linear solution, source modes, and frozen backbone carry reusable structure. It does not indicate an intrinsically smaller target problem.

#### 4.3.2. Probabilistic Accuracy and Calibration

**Figure 10:**
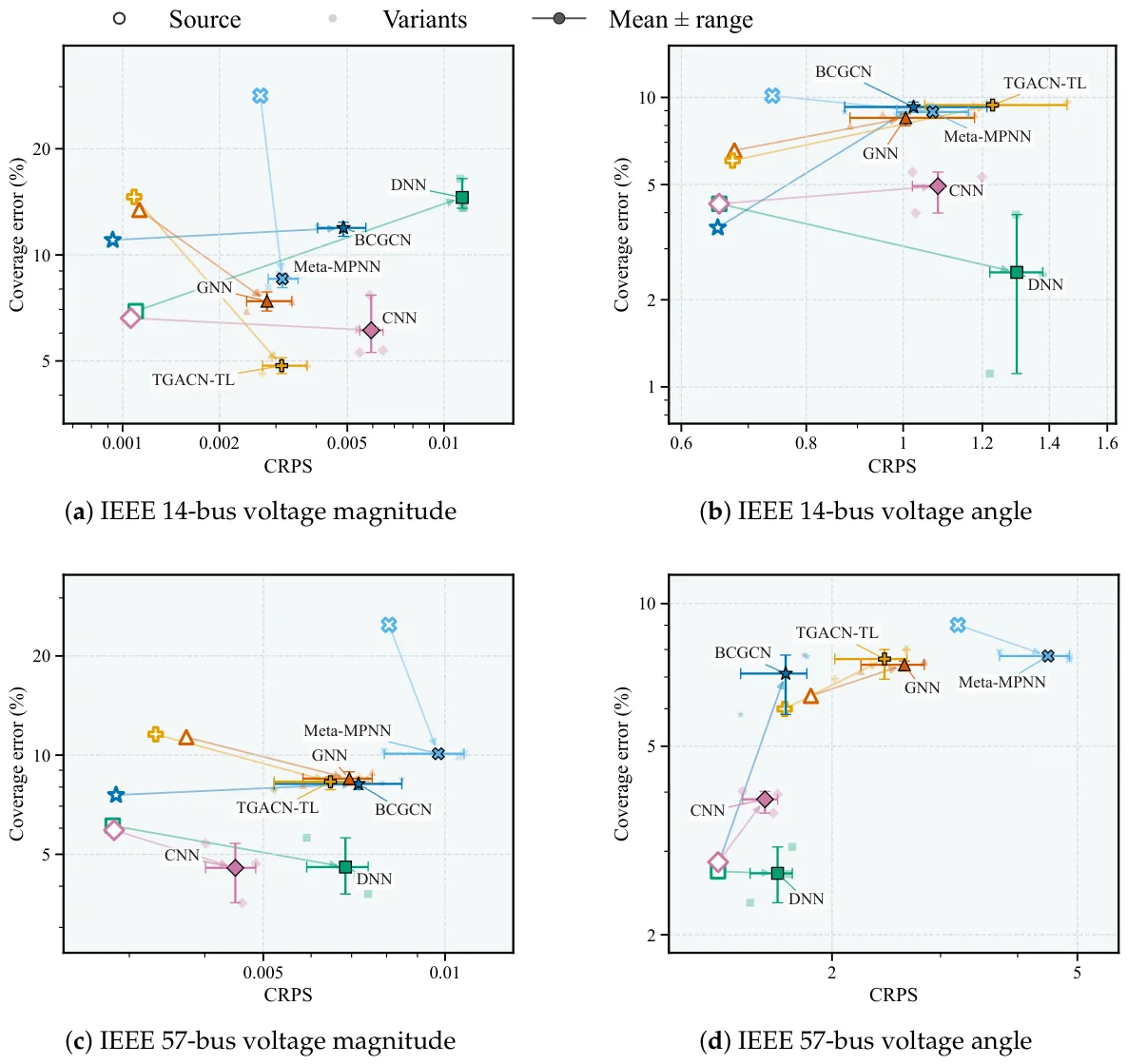
CRPS–coverage trade-off. Panels show the stated system and state variable; both axes use logarithmic scales, and lower-left is better. Open markers denote the source topology; faint markers show the three four-bus attachments; filled markers and whiskers show their mean and min–max range. Arrows connect the source value to the expanded mean.

Pointwise quantile errors do not assess distributional accuracy or calibration. We therefore evaluate CRPS, coverage, and monotonicity for the same six checkpoints. Figure 10 also tests attachment sensitivity: it shows the primary expansion and two one-parent alternatives per small system under matched settings. Open, faint, and filled markers denote the source, expanded variants, and their mean; whiskers show the variant range.

Before expansion, BCGCN has the lowest IEEE 14 CRPS ($9.32\times 10^{-4}$ p.u. for voltage and $0.652^{\circ }$ for angle) and is within 1.3% and 0.05% of the IEEE 57 minima. Its analytical baseline removes first-order AC response; the hybrid basis and gated complement then model structured and out-of-subspace residuals. This residual formulation explains its IEEE 14 CRPS lead without updating the full backbone.

After expansion, BCGCN recomputes the baseline and graph operator but updates only its 3.35% topology interface. It retains the four pointwise-error leads, yet its mean voltage/angle CRPS rises by 5.2/1.6 on IEEE 14 and 2.5/1.3 on IEEE 57. New branches introduce residual directions beyond the retained modes; the frozen ChebConv can reposition, but not relearn, its features. This degrades distributional calibration even when central quantiles remain accurate.

Accordingly, BCGCN angle coverage error rises from 3.55% to 9.27% on IEEE 14 and from 2.81% to 7.12% on IEEE 57; CNN has lower IEEE 57 voltage CRPS and coverage error, and DNN has lower angle coverage error. Coverage summarizes five intervals, whereas CRPS assesses the full curve. Thus BCGCN’s localized update gives the best pointwise quantiles in this regime, but expansion requires explicit recalibration. No condition shows adjacent-quantile crossing.

#### 4.3.3. Component Contributions

**Figure 11:**
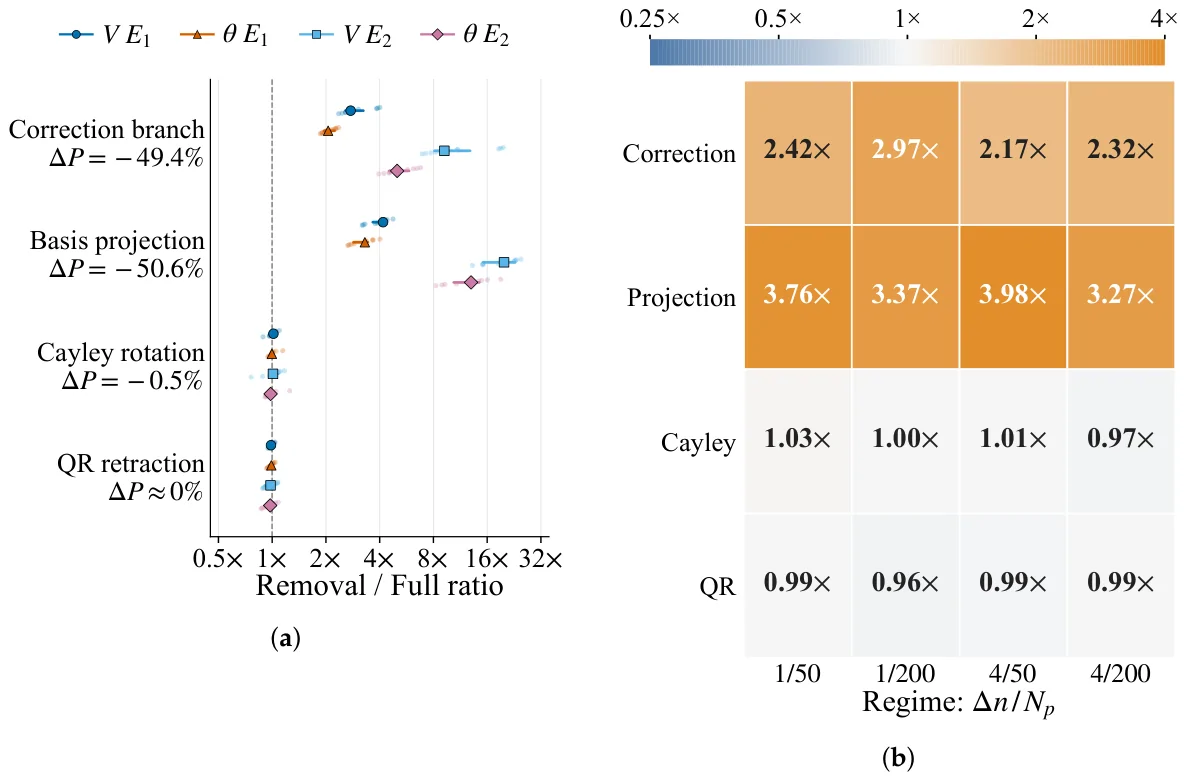
Few-shot component ablation. (**a**) Removal-to-Full ratios across 12 matched runs; markers and segments show medians and interquartile ranges. (**b**) Median joint $E_{1}$ ratio across transfer regimes (n=3 seeds); one denotes parity. Omitting QR retraction raises median $\epsilon_{⊥}$ from $6.75\times 10^{-8}$ to $8.85\times 10^{-2}$.

The ablation study uses 12 matched runs per removed component: two expansion sizes, two pilot-set sizes, and three random seeds. Removing the basis-projected branch causes the largest loss, with median ratios of 4.18 and 3.30 for $E_{1}^{V}$ and $E_{1}^{\theta }$ and 19.89 and 12.99 for their squared-error counterparts, as shown in Figure 11. Removing the orthogonal correction is consistently second, with median ratios ranging from 2.06 to 9.21 across the four output–metric pairs. The low-rank branch therefore provides the primary residual coordinates, whereas the complementary branch carries error components that cannot be discarded without a systematic penalty.

These two removals test complementary failure modes. Without the projected branch, the remaining correction is explicitly restricted by $I-QQ^{⊤}$ and cannot represent residual energy lying inside the hybrid subspace; the model loses the very coordinates in which topology-smooth and recurrent POD patterns were concentrated. Without the correction branch, the prediction is instead confined to span(Q), so truncation error and operating-point-dependent directions outside the retained modes cannot be recovered. The much larger squared-error ratios after projection removal show that this failure is not limited to a small uniform bias: large misses are amplified especially strongly. The ordered ablation effects are therefore structurally consistent with the direct-sum design of the residual, rather than being interchangeable regularization benefits.

The contribution hierarchy is stable across the four transfer regimes: the joint ratio is 3.27–3.98 without projection and 2.17–2.97 without the correction branch. Rotation and QR removal remain near ratio 1 in these local predictive tests, so their value should not be inferred from average error alone. QR removal instead changes the median effective-basis orthogonality error from $6.75\times 10^{-8}$ to $8.85\times 10^{-2}$, showing that the retraction enforces the stated basis constraint even when short-horizon prediction error is insensitive to it. The evidence thus distinguishes the components that dominate accuracy from those that preserve the geometry required by the adaptation formulation.

Stability across expansion size, pilot count, and seed strengthens this interpretation. Projection removal remains the largest effect in every regime, so the hybrid coordinates are useful even when more target samples are available; additional data do not let the complementary branch reconstruct a component that its projector removes. Conversely, the correction branch retains second-order importance across regimes, indicating that neither Laplacian modes nor empirical POD modes alone span the complete nonlinear response after expansion. Their roles are therefore hierarchical: the basis captures the dominant transferable residual, and the gated complement repairs systematic basis insufficiency.

The near-parity rotation and QR results answer a different question. A local bus addition initialized from source modes may require only a small within-subspace rotation, allowing the readout to compensate when the Cayley map is removed. Similarly, an unconstrained raw basis can fit short pilot runs even while its columns become correlated or rescaled. Predictive parity in these conditions does not establish that the operations are redundant. The four-order increase in $\epsilon_{⊥}$ after QR removal directly verifies loss of the stated Stiefel constraint.

#### 4.3.4. IEEE 118 Bus PV Variability Test

**Figure 12:**
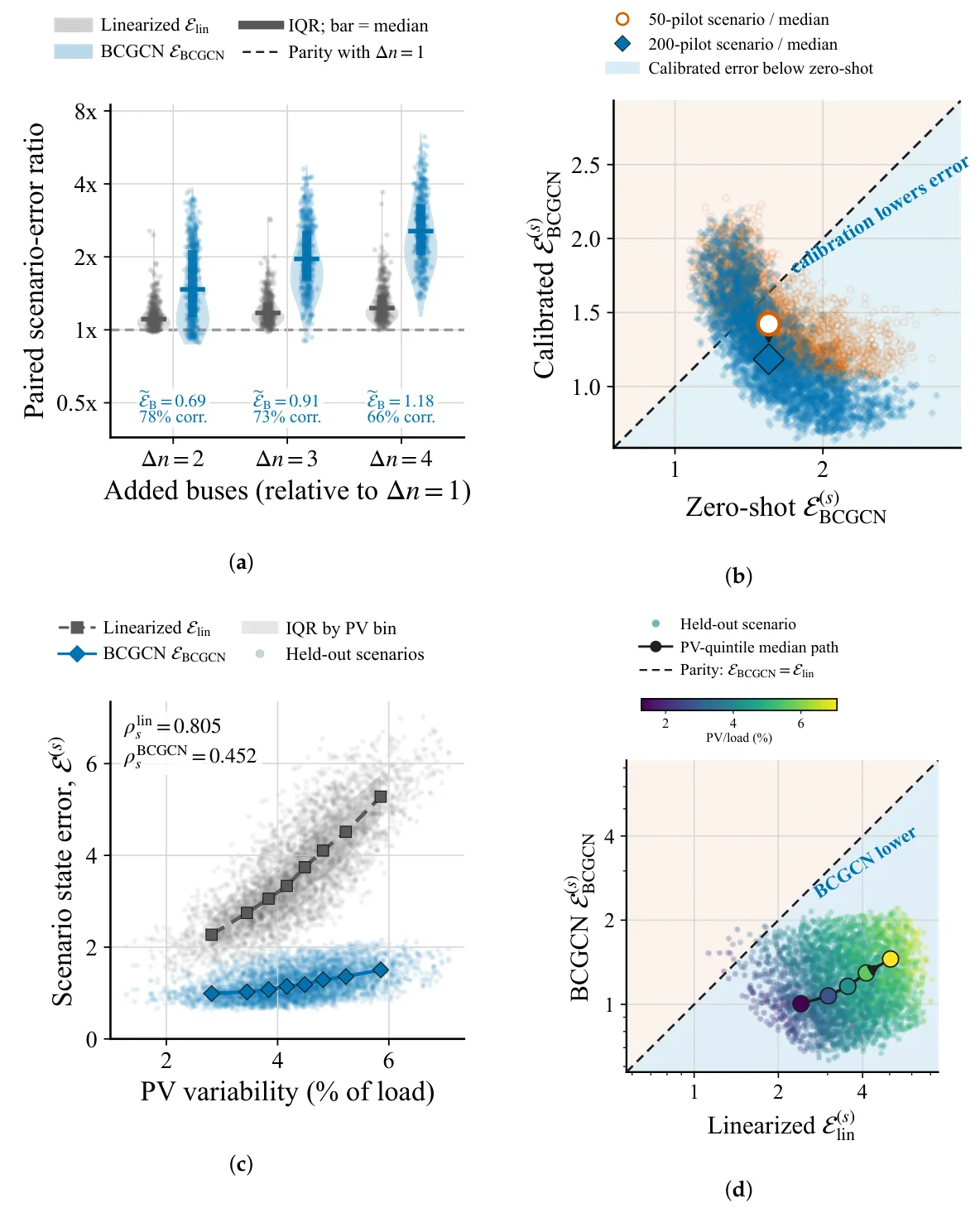
IEEE 118-bus scenario-level adaptation. (**a**) Growth ratios relative to Δn=1 across 3128 scenarios. (**b**) Zero-shot and 50/200-pilot recovery at Δn=4. (**c**) Errors versus PV variability. (**d**) Paired linearized and BCGCN errors by PV quintile.

The six-method results above compare accuracy across systems. This complementary IEEE 118 analysis compares BCGCN with the linearized solution at the scenario level under stronger renewable variability. It tests whether the residual correction remains useful after the common comparison has shown the loss of the BCGCN accuracy lead. Each condition contains the same 3128 test scenarios. With 200 pilot samples, the median $E_{\mathrm{BCGCN}}$ rises smoothly from 0.461 at one added bus to 1.185 at four, while $E_{\mathrm{lin}}$ rises from 2.843 to 3.535, as shown in Figure 12a. Size normalization changes the BCGCN endpoints from 0.0090 to 0.0229, confirming that the increase is not only a consequence of stacking more state variables. Nevertheless, BCGCN still removes 66.5% of the median linearization error at Δn=4, compared with 85.4% at Δn=1. The error is therefore bounded and progressive, but not topology size invariant.

The normalized trend separates representation difficulty from simple vector length. If the increase came only from concatenating more voltage and angle entries, division by $\sqrt{22n_{s}}$ would largely remove it. Its persistence shows that added buses change the per-coordinate prediction problem as well. Mechanistically, each tie line perturbs the target sensitivity and graph modes, while the transferred basis keeps the same source anchors and modal rank. The new rows and correction branch can absorb a local mismatch, but the fraction of linearization error removed falls as four such perturbations accumulate. This scale test therefore supports the same bounded-transfer interpretation as IEEE 57 without implying graph-size invariance.

Few-shot calibration recovers a material part of the four-bus expansion loss. The median BCGCN norm is 1.634 without target-topology calibration, 1.423 with 50 pilot scenarios, and 1.185 with 200, as shown in Figure 12b. Fifty scenarios deliver 46.8% of the total 0-to-200-pilot reduction; 200 scenarios reduce the zero-shot error by 27.5%. The recovery is therefore already visible in the smallest calibrated condition and continues as the pilot set grows.

The zero-shot value indicates that recomputing the analytical baseline and carrying source modal coordinates already transfer useful structure before a target label is used. Calibration then estimates where the new buses sit in that representation. The first 50 scenarios account for nearly half of the total measured improvement, which is consistent with a low-dimensional interface requiring fewer observations than a full backbone update. The remaining 150 scenarios deliver the other 53.2%, showing that the adapter has not saturated at 50 samples and still benefits from broader operating-point coverage.

PV variability amplifies the analytical approximation more strongly than the learned residual model. From the lowest to the highest variability quintile, $\nu_{\mathrm{PV}}$ increases from 3.04% to 5.63% of load. The median linearized error grows from 2.408 to 5.036 (2.09-fold), whereas the BCGCN error grows from 1.006 to 1.453 (1.45-fold). Spearman correlation with variability is 0.805 for $E_{\mathrm{lin}}$ and 0.452 for $E_{\mathrm{BCGCN}}$. Even in the highest quintile, the nonlinear residual correction removes 71.0% of the median linearization error. Across all paired scenarios BCGCN is better in 99.55% of cases, and in 100% of the highest-variability cases. Panels (c) and (d) therefore show both sides of the result: larger PV width exposes the expected linearization error, while the residual pathway substantially attenuates, rather than eliminates, its growth.

This attenuation follows the decomposition used by BCGCN. Increasing PV width moves scenarios farther from the nominal point, so neglected higher-order AC terms grow and the first-order sensitivity model deteriorates. The POD modes are estimated from nonlinear residual snapshots and can encode recurrent curvature that is absent from the Jacobian, while graph modes organize how that correction propagates over electrically connected buses. Residual directions not retained by either basis component remain available to the orthogonal correction. The reduction in Spearman correlation from 0.805 to 0.452 is therefore consistent with removal of a systematic variability-dependent component rather than a uniform rescaling of the linear error.

The paired comparison provides a stronger diagnostic than the quintile medians alone. Improvement in 99.55% of all scenarios, including every case in the highest quintile, rules out an average gain caused by sacrificing the most variable states. At the same time, BCGCN error still rises 1.45-fold and 29.0% of the highest-quintile median linearization error remains. The learned residual is thus robust to the tested PV range but does not remove operating- point dependence. Extrapolation beyond the observed variability range would require new evidence because both POD energy concentration and the linear anchor are distribution dependent.

### 4.4. Computational Cost

BCGCN couples the smallest adaptation-step time with competitive inference latency, as shown in Table 3. On the IEEE 14-bus expansion, its 1.88 ms median inference time is the lowest among the six methods. On the IEEE 57-bus expansion, DNN has the lowest median at 1.87 ms, but BCGCN has the lower 95th percentile (4.21 versus 4.92 ms) and the highest measured batch-64 throughput, 29,213 samples/s. BCGCN also reaches 28,372 samples/s on the IEEE 14-bus expansion.

Inference latency reflects the forward operator, not the number of trainable parameters. DNN benefits from dense GPU kernels and therefore has the lowest IEEE 57 median, whereas edge-conditioned GNN, TGACN-TL, and Meta-MPNN perform edge gathering and aggregation at every message-passing layer. BCGCN adds a linear baseline and modal reconstruction, but its graph-size-independent Chebyshev backbone and low-dimensional coefficient path keep the median close to the dense model while producing the smallest reported 95th percentile on IEEE 57. The higher batch throughput is consistent with that compact residual path amortizing fixed operations across scenarios.

The adaptation step isolates the practical benefit of freezing the backbone. BCGCN requires 3.44 ms on the IEEE 14-bus expansion and 4.78 ms on the IEEE 57-bus expansion, versus 8.74 and 8.51 ms for the next-fastest DNN adaptation.

The adaptation-step gap has a different mechanism from inference. Freezing the backbone removes its gradient tensors and Adam updates, so only 3.35% of BCGCN’s parameters participate in backpropagation. The DNN exposes every weight despite its inexpensive forward pass; accordingly, its adaptation step is 2.54 times slower on IEEE 14 and 1.78 times slower on IEEE 57. GNN, Meta-MPNN, and CNN also update their full networks, while TGACN-TL updates a larger 38.98% transfer block and retains attention operations. The timing order is therefore consistent with localized optimization reducing backward and optimizer work, whereas forward latency remains governed by the operators executed by each architecture.

## 5. Conclusions

BCGCN treats local node addition as a constrained modal interface update rather than a full relearning problem. A linearized solution supplies the first-order response. Topology-weighted Laplacian and residual POD modes represent graph geometry and recurrent nonlinear structure. A gated orthogonal complement captures residuals outside the retained basis. After expansion, new bus rows, QR retraction, and Cayley rotation update this interface while the ChebConv backbone remains frozen.

BCGCN is designed for small-scale grids with local additions. The common six-method results establish both its operating range and its boundary. It ranks first in all four mean and 0.95 quantile errors on the IEEE 14 and IEEE 57 four bus expansions. At 200 pilot samples, the best baseline mean voltage and phase-angle errors are 2.42 and 3.79 times the BCGCN values on IEEE 14 and 1.81 and 6.59 times the values on IEEE 57. IEEE 118 identifies the transition out of this range. BCGCN wins 9 of the 64 error cells across the complete IEEE 118 grid, all in voltage metrics with one or two added buses. It has no four0metric lead after four additions, although it updates only 5820 parameters compared with 0.333 to 9.041 million for the baselines. The complete Polish grid supplies the large network limit. BCGCN updates 5859 parameters but does not lead a reported error cell. It therefore remains economical to update at large scale, but it is not an accuracy-preserving adapter for large networks.

The ablation and IEEE 118 PV results explain why the compact interface remains useful within its intended range. Removing basis projection or complementary correction raises the median joint $E_{1}$ ratio to 3.27 to 3.98 or 2.17 to 2.97. The projected residual carries the main signal and the complement repairs missing directions. QR removal leaves short horizon prediction near parity but changes effective basis orthogonality error from $6.75\times 10^{-8}$ to $8.85\times 10^{-2}$. QR and Cayley therefore preserve and adjust transfer geometry rather than guaranteeing lower average error on their own. On IEEE 118, 50 pilots recover 46.8% of the zero-shot-to-200-pilot improvement, 200 pilots reduce zero-shot error by 27.5%, and residual correction removes 71.0% of the highest-quintile median linearization error. Median loaded checkpoint inference and adaptation step latencies are 1.88 to 2.08 ms and 3.44 to 4.78 ms.

The evidence is limited to local additions in balanced networks, historical PV uncertainty, and offline AC Monte Carlo labels. The CRPS and coverage results refine this boundary. BCGCN attains the lowest source-topology CRPS for both IEEE 14 state variables and remains close to the IEEE 57 minima because its analytical baseline and hybrid residual representation capture the dominant source distribution. After expansion, however, its localized interface update preserves the pointwise-error advantage without uniformly minimizing CRPS or coverage error. Low marginal quantile errors therefore do not guarantee the best-calibrated distribution after a topology change, and explicit recalibration is needed. Future work should add an explicit calibration mechanism, increase or adapt modal capacity for larger target grids, and then test line switching, unbalanced feeders, delayed measurements, cyber physical disturbances, severe power deficits, and EMS, OPF, and contingency settings under more extreme renewable conditions before claiming operational robustness.

## Figures and Tables

**Table 1:** Core method configurations; *Adapted* lists parameters updated on the pilot set.

Method	Core Predictor	Structural Interface	Adapted Parameters	Essential Setting
BCGCN	Chebyshev residual GCN	Linear AC + hybrid basis	New-bus basis, Cayley rotation, readouts, correction gate	4×128; K=3
GNN	Edge-conditioned MP	Electrical graph	Full network	4×128
DNN	Dense mapping	None	Full network	4×128
CNN	1-D convolution	Fixed bus order	Full network	4×128
TGACN-TL	Topology attention	Graph attention	Transfer block + head	Partial transfer
Meta-MPNN	Edge-conditioned MP	Meta-initialization	Full network	MAML

**Table 2:** Polish 2746-bus source-topology results from one common held-out split. Pinball loss and $E_{1}^{V}$ are reported in units of $10^{-3}$; $E_{1}^{\theta }$ is in degrees. Tail errors for the same checkpoints are shown in Figure 7c.

Method	Trainable Parameters (M)	Pinball Loss	$E_{1}^{V}$ (p.u.)	$E_{1}^{\theta }$ (deg)
BCGCN	0.171	2.041	0.835	1.302
GNN	0.620	6.221	1.217	3.891
DNN	182.015	1.783	0.716	1.154
CNN	181.449	1.850	0.869	1.158
TGACN-TL	0.853	5.418	0.882	3.321
Meta-MPNN	0.620	14.723	26.132	7.962

**Table 3:** GPU runtime from loaded checkpoints: median/95th-percentile milliseconds for single-scenario inference and one in-memory adaptation step.

Method	IEEE 14-Bus +4 Buses		IEEE 57-Bus +4 Buses	
	Inference	Adaptation Step	Inference	Adaptation Step
BCGCN	1.88/3.99	3.44/6.87	2.08/4.21	4.78/5.74
GNN	5.86/6.71	14.67/17.72	6.00/7.18	14.69/17.68
DNN	2.04/4.87	8.74/11.62	1.87/4.92	8.51/11.79
CNN	2.52/5.31	13.45/18.73	2.11/5.40	12.32/18.46
TGACN-TL	7.35/9.39	14.24/17.01	7.20/8.86	14.46/17.19
Meta-MPNN	5.70/6.51	13.97/17.61	5.83/6.51	14.28/17.98

## Data Availability

The data presented in this study are available on request from the corresponding author due to privacy restrictions.
